# Investigating Patient Trust and Intention to Act in AI-Supported Clinical Decisions: Cross-Sectional Survey

**DOI:** 10.2196/100388

**Published:** 2026-11-02

**Authors:** Sippo Rossi, Afra Shamnath, Meghan Reading Turchioe, Laurie Lovett Novak, Megan E Salwei, Isabella Buitron, Xuhai Xu, Natalie Benda

**Affiliations:** 1 Department of Management and Organisation, Hanken School of Economics, Arkadiankatu 22, Helsinki, Uusimaa, 00100, Finland, 358 503131343; 2 School of Nursing, Columbia University, New York, NY, United States; 3 Department of Biomedical Informatics, Vanderbilt University Medical Center, Nashville, TN, United States; 4 Department of Biomedical Informatics, Columbia University Irving Medical Center, New York, NY, United States

**Keywords:** artificial intelligence, risk communication, patient-centered care, clinical decision-making, shared decision-making

## Abstract

**Background:**

In health systems, AI is increasingly being implemented as a form of clinical decision support (CDS). Research on patient perspectives, however, lags behind clinician-focused studies. Before the introduction of AI-based tools, clinicians often used and continue to use rule-based guidelines and risk calculators (RCs) to inform decision-making. Prior research has shown that RCs for CDS have mixed effects on patient adherence to treatment recommendations, whereas for AI-based tools, the effect is still not clear.

**Objective:**

This study aims to assess differences in patients’ behavioral intentions, trust, and preferences for clinical recommendations provided by doctors alone, doctors supported by RCs, and doctors supported by AI-based risk predictions. We also evaluate how sociodemographic characteristics affect these perceptions.

**Methods:**

We surveyed 1185 US-based adults. Participants rated their likelihood of taking recommended clinical actions (behavioral intentions) across 6 clinical scenarios, where they were randomized to a different recommendation source (doctor, doctor + RC, and doctor + AI). Participants also rated their trust in the recommendation, indicated their preferred source for recommendations, and provided qualitative feedback.

**Results:**

Behavioral intentions differed by recommendation source such that the doctor + RC condition had greater odds of behavioral intention than the doctor + AI condition. Behavioral intentions were also affected by the clinical condition. Participants had greater odds of trusting doctors alone over doctor + RC, and both doctors alone and doctor + RC had greater trust than doctor + AI. However, a plurality of participants ultimately preferred a doctor using an RC for making recommendations. Several sociodemographic differences were detected, namely, participants with higher subjective numeracy and literacy had higher odds of taking clinical action and higher trust across decision-support sources. We detected qualitative themes in the following categories: decision quality and reliability; trust and perceptions; patient-centeredness and autonomy; fairness, transparency, and ethics. Across categories, participants reported skepticism toward all decision sources and potential misunderstanding of AI’s ability to personalize risk predictions. Some participants also stated that they had no additional concerns or were uncertain regarding their feelings.

**Conclusions:**

These findings reveal novel, nuanced patient preferences shaped by task and context, offering guidance for aligning AI-supported care with patient values. Patients were not aligned on the recommendation source across intentions, trust, and preferences. Taken together, our results indicate that patients prefer doctors working with easy-to-understand, explainable support but are still learning to trust these tools. Our findings also highlight the continued importance of the clinical context and its interaction with computer-assisted decision support. Future work should evaluate how we can transparently convey to patients the use of AI in clinical decision-making, embedded within the given clinical context. This may present an opportunity to bolster, rather than erode, patient trust.

## Introduction

### Background and Significance

Recent media coverage of AI has centered around generative large language models and the burgeoning use of agentic AI. In health systems, AI is being increasingly implemented as a form of clinical decision support (CDS), supporting clinician judgment by estimating risk levels, interpreting images, or informing treatment planning [1-5].

It is more likely than ever that patients may encounter AI risk scores due to initiatives such as the US 21st Century Cures Act and OpenNotes [6-8]. Research on patient perspectives, however, lags behind clinician-focused studies [9]. Patients may now encounter AI-mediated information directly and therefore deserve clear, comprehensible explanations of how AI systems generate predictions that may affect their care [10].

Despite an imbalance in the focus on clinician perspectives on AI supporting clinical decision-making [9], recent studies have elicited more patient perspectives [11,12]. Our previous work indicates the importance of keeping patients informed about AI-derived risks to justify treatment decisions and promote adherence [13]. Several additional studies by our team and others have explored patient trust and reliance on decisions made by AI alone or solely by clinicians [11,12,14-20]. To date, however, there are few clinical scenarios where AI would make decisions and act independently without a clinician in the loop. Therefore, comparing differences in perceptions of AI-assisted clinical decision-making to human decision-making alone is more pertinent [21].

Before the introduction of machine learning–based AI tools in health care, clinicians often used, and also continue to use rule-based guidelines and risk calculators (RCs) to inform decision-making [22-24]. However, the RCs for CDS have shown mixed effects on patient adherence to treatment recommendations [25-28]. Therefore, we aim to understand if perceptions differ when clinicians are assisted with these rule-based tools, using RCs as an example, as opposed to the potentially more powerful but less readily explainable AI-based tools.

Finally, our previous work has also shown that trust and reliance on AI-informed decisions depend on the clinical prediction task [11,12,14,15]. Systematic reviews suggest that patients are less accepting of AI tools applied to “more serious or sensitive health conditions” [11]. While previous studies typically focus on a singular clinical decision, we explore differences across a series of clinical applications where RCs and AI may provide CDS, but that also have elements of serious or sensitive disease.

### Objectives

We evaluate patient perspectives on AI tools supporting CDS for 6 different clinical conditions, with attention to differences across sociodemographic groups. Our research questions included as follows:

1. Does a patient’s propensity to accept/trust in clinician recommendations differ based on whether the decision is supported by an RC, AI, or neither?
2. Does a patient’s propensity to accept/trust clinician recommendations vary based on sociodemographic characteristics?

We also summarize open-ended participant feedback regarding perspectives on RC and AI-based CDS to contextualize our quantitative findings.

## Methods

### Study Design and Participant Recruitment

We conducted a cross-sectional online survey of US-based adults between June 17, 2024, and July 26, 2024. We recruited participants via Qualtrics’ survey sampling service, which drew respondents to the closed survey from an opt-in online panel. Eligibility criteria included being at least 18 years of age, being able to read English, and providing informed consent within the Qualtrics platform before beginning the survey.

No personal information that would allow identification of participants was collected during the survey. To support comparisons across sociodemographic groups, we used quota sampling balanced on sex, education (with vs without a bachelor’s degree or higher), and generation (Baby Boomer, Generation X [Gen X], Millennial, and Generation Z [Gen Z]). We also targeted approximately half of the sample to identify as members of racial or ethnic minority groups. Participants were given monetary compensation for participating in the survey.

### Ethical Considerations

The Columbia University Institutional Review Board approved this study (IRB review number: AAAU3382). The design and results of the survey are reported in accordance with the CHERRIES (Checklist for Reporting Results of Internet E-Surveys) guidelines (Checklist 1) [29].

### Survey Development

We developed survey items with input from experts in human factors, anthropology, human-centered AI, AI development, nursing, primary care, and survey design. Our team iteratively drafted and refined the items through multiple rounds of pilot testing with both experts and laypersons to assess comprehensibility and face validity. Excluding unavoidable technical terms (eg, “artificial intelligence” and clinical vocabulary), we targeted an eighth-grade reading level. The full survey battery of questions used during data collection is provided in Multimedia Appendix 1. The results of the generative AI questions that appear in the survey are not included in this paper.

### Procedure

After confirming age eligibility and obtaining consent, demographic questions were used to determine whether respondents met current quota needs. Those proceeding to the survey received definitions of “risk calculators” and “artificial intelligence,” and confirmed their understanding. Definitions stated:

- *Risk calculators allow doctors, nurses, and patients to enter information about a patient (such as, age, gender, smoking status) and calculate a number that shows a patient’s risk, for example, of having a stroke (see picture below). These tools can easily explain how they get to the risk prediction because they use simple math*.
- *Artificial intelligence (AI) or “AI,” refers to computer programs that can learn to make decisions or predictions like humans using large amounts of data. AI, for example, may take large amounts of data from patients they know did and did not have a stroke in their lifetime. The AI learns patterns from this data and predicts who is at high risk of stroke in the future. AI is different from a risk calculator in that it cannot always explain the rules it used to make a prediction*.

Participants were asked about their behavioral intentions, trust, and preferences regarding medical decisions made by (1) a doctor alone, (2) a doctor supported by an RC, and (3) a doctor supported by AI. Each participant viewed six clinical scenarios (lung cancer, sepsis, depression, colon cancer, respiratory syncytial virus [RSV] vaccination, and heart failure). Participants were randomly assigned to 1 of 6 blocks, which included each decision source in 2 of the 6 scenarios and each clinical condition once, with no decision source repeated immediately on consecutive scenarios. The order of clinical conditions and decision sources was partially balanced to mitigate ordering effects. This process is illustrated in Textbox 1.

Textbox 1. **Description and example of the scenarios seen by participants**

**Sources of medical decision (each encountered twice by participants):**

- Doctor
- Doctor + risk calculator
- Doctor + AI

**Clinical scenarios (each encountered once by participants)**

- Lung cancer
- Colon cancer
- Sepsis
- Depression
- Heart failure
- Respiratory syncytial virus (RSV) vaccination

**Example of scenarios seen by participant (combinations shown in randomized order)**

- Doctor + AI, lung cancer
- Doctor + risk calculator, colon cancer
- Doctor, depression
- Doctor + AI, heart failure
- Doctor + risk calculator, sepsis
- Doctor, RSV vaccination

Scenarios were selected to represent contexts where AI-based CDS may be viable and to vary in seriousness and sensitivity, given prior findings that patients may be less comfortable with AI for serious or sensitive conditions [14]. Each scenario included a brief description of the condition (adapted from MedlinePlus patient education materials) [30], followed by items assessing intended behavior and trust in the recommendation (Table 1).

**Table 1.** AI clinical decision support (CDS) task scenarios, clinical decisions, and elements rationalizing selection.

Condition	Clinical decision	Serious or sensitive content element (rationale for inclusion)
Lung cancer	Deciding whether or not to get a lung biopsy based on possible cancer detected on a chest x-ray	Serious disease (also involved AI for imaging)
Colon cancer	Deciding whether or not to have a colonoscopy based on high personal risk of colon cancer	Serious disease
Sepsis	Deciding whether or not to proceed with treatment for sepsis warning signs for a loved one admitted to the intensive care unit.	Serious disease, proxy decision-making
Depression	Deciding whether or not to receive a psychiatric evaluation based on risk factors for depressive disorder	Sensitive condition (mental health)
Heart failure	Deciding whether or not to be discharged from the emergency room (instead of admitted) for predicted low-risk heart failure exacerbation	Serious disease, low-risk prediction
RSV^a^	Deciding whether or not to get an RSV^a^ vaccine based on higher than average personal risk factors	Serious disease, sensitive treatment (due to vaccine hesitancy)

a RSV: respiratory syncytial virus.

After completing all scenarios, participants indicated their preferred CDS approach (doctor alone, doctor + RC, or doctor + AI) for each condition. Participants were then invited to provide qualitative feedback about their concerns regarding each decision-support approach.

Before completing the survey, participants answered additional demographic measures on subjective numeracy (3-item Subjective Numeracy Scale) [31], health literacy (3-item Brief Health Literacy Screen) [32], perceived financial stability, comfort with the use of technology, and perceived discrimination in medical settings (7-item Discrimination in Medical Settings scale) [33].

### Power Calculation

We powered the study to detect differences in behavioral intentions across the 3 CDS conditions (doctor alone, doctor + RC, doctor + AI). Based on prior AI-related acceptability studies, we considered a 15% absolute difference (approximately a medium effect size; odds ratio [OR] 1.94) to be practically significant (*α*=.05; *β*=.80). Using these parameters in R (version 4.5.0; R Foundation for Statistical Computing), we estimated a minimum of 256 participants per decision-support group (n=768). We set 768 as the minimum target and allowed additional enrollment to facilitate subgroup analyses.

### Data Analysis

#### Quantitative Analysis

All quantitative analyses were conducted using R (versions 4.5.0 and 4.6.1) and IBM SPSS (version 29). We first computed descriptive statistics to assess distributions, skew, missingness, and potential outliers. Because the proportional odds assumption was violated for behavioral intention and trust (both *P*<.01), we then used multinomial (rather than ordinal) logistic regression for these outcomes. For both outcomes, we compared differences in participants reporting they were negative vs neutral and neutral vs positive based on decision-support approach (doctor, doctor + RC, and doctor + AI), clinical condition, and sociodemographic variables.

We also estimated a multinomial model for the preferred decision-support approach. The assessed sociodemographic variables were consistent across models and included gender, race (White vs Black/African American or Asian), health literacy (categorized per instrument guidance) [32], numeracy (continuous), discrimination in medical settings (continuous), and generation. Our analyses focused on racial groups constituting 5% of the total sample, rounded to the nearest percentage point. The generations were defined as Baby Boomer (born 1945‐1964), Gen X (1965‐1980), Millennial (1981‐1996), and Gen Z (1997‐2006) [34]. We assessed collinearity prior to modeling and confirmed that all variance inflation factors were <5.

#### Qualitative Analysis

We conducted a qualitative analysis of open-ended responses to three survey items in which participants were asked to describe any concerns regarding: (1) physicians (human), (2) RCs, and (3) AI-supported decision-making. Although the survey included additional open-ended fields, only these 3 items were included in the present analysis.

We used a directed content analysis approach, combining deductive and inductive coding. An initial codebook was developed based on prior work [14,35] and included a priori themes and subthemes, which were refined through inductive review of the current dataset. Each open-ended response was assigned a single code, and categories within each theme were mutually exclusive. Two authors independently coded a random subset of responses from 250 participants (encompassing more than 20% of the dataset) across the three decision-making conditions to establish consistency. Interrater reliability was assessed using Cohen κ, demonstrating substantial agreement (human: κ=0.642; RC: κ=0.618; AI: κ=0.600) [36,37]. Discrepancies were resolved through discussion, and the codebook was refined accordingly. The remaining responses were then divided, with each coder independently coding half of the dataset. Theme and subtheme frequencies were calculated for each decision-making condition (human, RC, AI).

## Results

### Participants

Table 2 describes the sociodemographic characteristics of 1185 participants who completed the survey.

**Table 2.** Reported demographic characteristics of participants (N=1185).

Characteristic	Frequency, n (%)
Sex at birth	
Male	585 (49.4)
Female	599 (50.5)
Do not know	1 (0.1)
Gender	
Man	580 (48.9)
Woman	599 (50.5)
Nonbinary/transgender	6 (0.5)
Race	
Black	491 (41.4)
White	600 (50.6)
Asian	57 (4.8)
American Indian or Alaska Native	19 (1.6)
Native Hawaiian or other Pacific Islander	3 (0.3)
Prefer not to answer	15 (1.3)
Ethnicity	
Not Hispanic/Latino	1122 (94.7)
Hispanic/Latino	51 (4.3)
Unsure/prefer not to answer	12 (1.0)
Age generation	
Boomer	388 (32.7)
Generation X	337 (28.4)
Millennial	292 (24.6)
Generation Z	168 (14.2)
Education	
Less than bachelor’s degree	585 (49.4)
Bachelor’s degree or more	598 (50.5)
Prefer not to answer	2 (0.2)
Numeracy	
Low	675 (57.0)
High	502 (42.4)
Unsure/prefer not to answer	8 (0.7)
Health literacy	
Low	625 (52.7)
High	550 (46.4)
Unsure/prefer not to answer	10 (0.8)
Financially stable	
More than enough	155 (13.1)
Enough	490 (41.4)
Not enough	510 (43.0)
Unsure/prefer not to answer	30 (2.5)
Discrimination in medical settings	
Low	604 (51.0)
High	571 (48.2)
Unsure/prefer not to answer	10 (0.8)

### Quantitative Findings

For each clinical condition, we examined whether the distribution of participants who were likely vs undecided and likely vs unlikely to take the recommended action differed by recommendation source (doctor, doctor + RC, or doctor + AI) and sociodemographic variables. Figure 1 shows the percentage likely, undecided, or unlikely to complete the recommended behavior based on recommendation source for each clinical condition. When viewing doctor + AI decision support, as compared to doctor + RC (reference), participants had lower odds of reporting their behavioral intention as likely (OR 0.84, 95% CI 0.72-0.99; Table 3) as opposed to unlikely. Participants with higher numeracy and literacy had higher odds of reporting likelihood for clinical action, while Gen Z had lower odds. We also found significant differences based on clinical condition such that participants reported lower odds of behavioral intention for the heart failure, depression, and RSV scenarios than for the sepsis scenario (reference). Participants also had lower odds of being “undecided” on behavioral intentions, compared to unlikely, for the colon cancer, depression, heart failure, and RSV conditions. Those with higher literacy also had greater odds of reporting their behavioral intentions as undecided, rather than unlikely.

**Figure 1.**
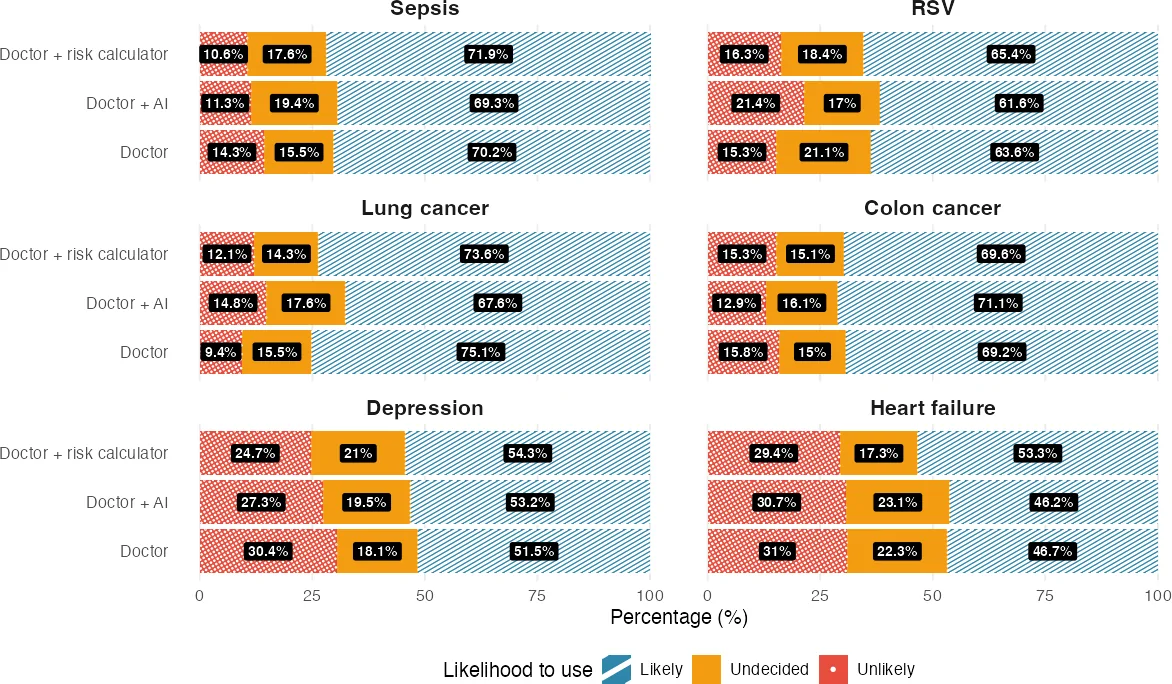
Behavioral intentions across clinical conditions. RSV: respiratory syncytial virus.

**Table 3.** Mixed-effects multinomial logistic regression for behavioral intentions^a^.

Predictor	Undecided vs unlikely		Likely vs unlikely	
	OR^b^ (95% CI)^c^	*P* value	OR (95% CI)	*P* value
Intercept	1.09 (0.79‐1.50)	.60	7.82 (5.97‐10.23)	<.001
Source (reference: doctor + risk calculator)				
Doctor	0.97 (0.79‐1.18)	.74	0.88 (0.75‐1.04)	.13
Doctor + AI	1.01 (0.83‐1.23)	.92	0.84 (0.72‐0.99)	.03
Condition (reference: sepsis)				
Lung cancer	0.87 (0.63‐1.19)	.38	1.02 (0.78‐1.32)	.89
Colon cancer	0.70 (0.52‐0.96)	.03	0.80 (0.62‐1.03)	.08
Depression	0.50 (0.38‐0.66)	<.001	0.29 (0.23‐0.37)	<.001
Heart failure	0.49 (0.37‐0.65)	<.001	0.24 (0.19‐0.30)	<.001
RSV^d^	0.73 (0.55‐0.99)	.04	0.58 (0.46‐0.74)	<.001
Women (reference: men)	1.10 (0.89‐1.35)	.38	0.96 (0.80‐1.16)	.69
Race (reference: White)				
Asian	1.09 (0.68‐1.73)	.73	1.03 (0.68‐1.56)	.89
Black	1.03 (0.84‐1.28)	.75	0.92 (0.76‐1.11)	.39
Generation (reference: Baby Boomer)				
Generation X	1.14 (0.89‐1.48)	.30	1.09 (0.87‐1.36)	.46
Millennial	1.15 (0.87‐1.52)	.31	1.06 (0.83‐1.36)	.62
Generation Z	0.81 (0.59‐1.11)	.20	0.61 (0.46‐0.81)	<.001
Numeracy (*z *score)	0.98 (0.88‐1.08)	.68	1.33 (1.21‐1.45)	<.001
Health literacy (*z *score)	1.26 (1.13‐1.42)	<.001	1.20 (1.08‐1.33)	<.001
Discrimination in medical settings (*z *score)	1.03 (0.92‐1.16)	.61	1.04 (0.93‐1.15)	.50

a Mixed effects multinomial logit model; reference outcome: unlikely. Random intercepts by participant: variance (undecided) 0.68, variance (likely) 0.89, covariance –0.10. Observations: 7012; participants included: 1169.

b OR: odds ratio.

c 95% CIs are Wald intervals.

d RSV: respiratory syncytial virus.

We then assessed differences in agreement with trust statements (eg, “I trust the doctor and AI to make a recommendation about a biopsy for lung cancer”), comparing distributions of participants agreeing vs being neutral, and agreeing vs disagreeing with the statements. Figure 2 displays results by condition. Participants had greater odds of trusting recommendations (agree vs disagree) made by their doctor alone compared to the doctor + RC (OR 1.35, 96% CI 1.13-1.61; Table 4), but lower odds of trusting the doctor + AI (OR 0.59, 95% CI 0.50-0.69; Table 4). Participants born in Gen X, those with higher numeracy, and higher literacy also had greater odds of trusting clinical recommendations, while women and those reporting higher discrimination in medical settings had lower odds. Compared to the sepsis scenario (reference), participants had greater odds of trusting recommendations regarding both cancers (lung and colon) but lower odds of trust for the depression and heart failure scenarios.

**Figure 2.**
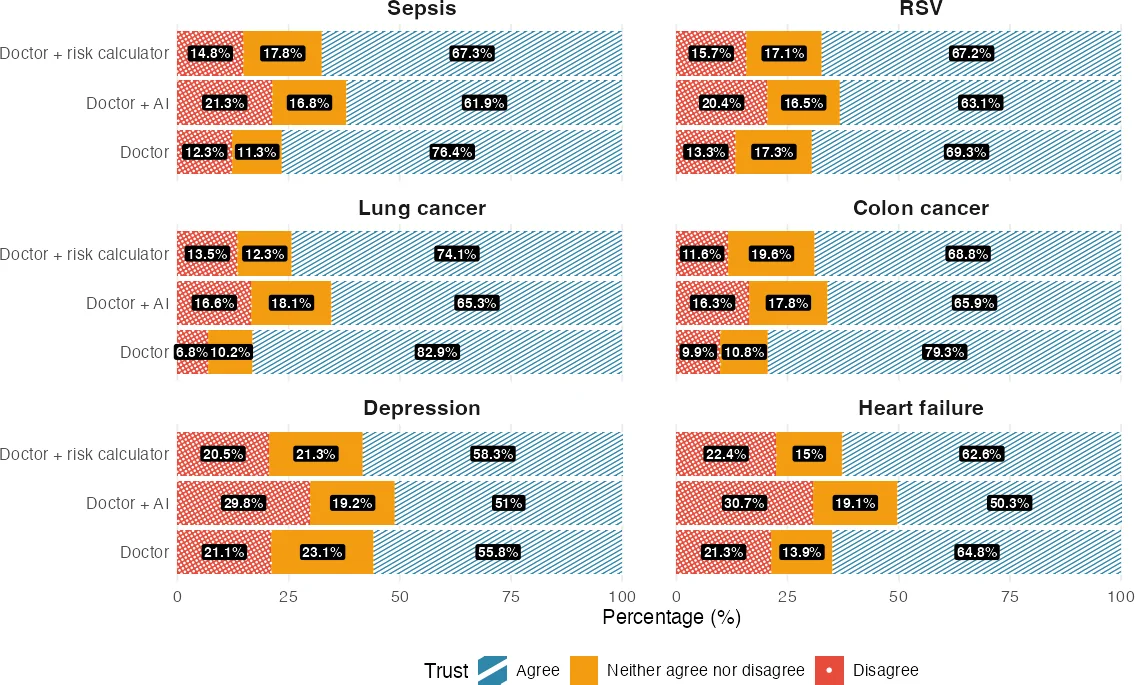
Trust across clinical conditions. RSV: respiratory syncytial virus.

**Table 4.** Mixed-effects multinomial logistic regression for trust^a^.

Predictor	Neutral vs disagree		Agree vs disagree	
	OR^b^ (95% CI)^c^	*P* value	OR (95% CI)	*P* value
Intercept	0.82 (0.58‐1.14)	.23	6.36 (4.73‐8.55)	<.001
Source (reference: doctor + risk calculator)				
Doctor	0.96 (0.77‐1.19)	.70	1.35 (1.13‐1.61)	.001
Doctor + AI	0.75 (0.62‐0.92)	.006	0.59 (0.50‐0.69)	<.001
Condition (reference: sepsis)				
Lung cancer	1.16 (0.84‐1.60)	.37	1.53 (1.18‐1.99)	.001
Colon cancer	1.38 (1.01‐1.89)	.045	1.40 (1.08‐1.82)	.01
Depression	0.95 (0.71‐1.26)	.72	0.44 (0.35‐0.56)	<.001
Heart failure	0.67 (0.50‐0.90)	.009	0.48 (0.38‐0.61)	<.001
RSV^d^	1.09 (0.81‐1.48)	.57	0.91 (0.71‐1.17)	.48
Women (reference: men)	0.91 (0.72‐1.15)	.42	0.74 (0.59‐0.93)	.009
Race (reference: White)				
Asian	1.13 (0.64‐1.97)	.68	1.48 (0.87‐2.52)	.15
Black	0.94 (0.74‐1.19)	.61	0.87 (0.69‐1.10)	.25
Generation (reference: Baby Boomer)				
Generation X	1.50 (1.12‐2.00)	.006	1.35 (1.02‐1.79)	.04
Millennial	1.56 (1.14‐2.13)	.006	1.35 (1.00‐1.84)	.050
Generation Z	1.01 (0.70‐1.44)	.97	0.80 (0.57‐1.14)	.21
Numeracy (*z *score)	1.06 (0.94‐1.19)	.36	1.49 (1.33‐1.67)	<.001
Health literacy (*z *score)	1.28 (1.13‐1.46)	<.001	1.18 (1.04‐1.34)	.01
Discrimination in medical settings (*z *score)	0.83 (0.73‐0.95)	.008	0.83 (0.73‐0.95)	.006

a Mixed effects multinomial logit model; reference outcome: disagree. Random intercepts by participant: variance (neutral) 1.02, variance (agree) 1.78, covariance 0.24. Observations: 7014; participants included: 1169.

b OR: odds ratio.

c 95% CIs are Wald intervals.

d RSV: respiratory syncytial virus.

Participants also had lower odds of neutrality for feelings of trust, compared to distrust (disagree) for the doctor + AI than the doctor + RC condition. Gen X, Millennials, and those with higher literacy also had greater odds of neutrality, while those experiencing discrimination in medical settings had higher odds of distrust. Compared to the sepsis scenario (reference), participants had greater odds of neutrality in the colon cancer scenario but lower odds in the heart failure scenario.

Figure 3 shows preferences for the recommendation source by condition. A plurality (n=540-627, 46.2%‐53.6%) preferred doctor + RC in all conditions except depression, where a narrow plurality preferred doctor alone (n=492, 42.1%) over doctor + RC (n=468, 40.0%). Doctor + AI was the least preferred, with values ranging from 17.9% (n=209) to 21.1% (n=247). Multinomial logistic regression models identified significant omnibus effects for heart failure (*P*=.009), depression (*P*=.049), sepsis (*P*=.01), and RSV (*P*<.001).

The full results of the multinomial logistic regressions shown in Figure 3 are provided in the Multimedia Appendix 2. Additional data for the figures, as well as subgroup sample sizes, are provided in Multimedia Appendix 3.

**Figure 3.**
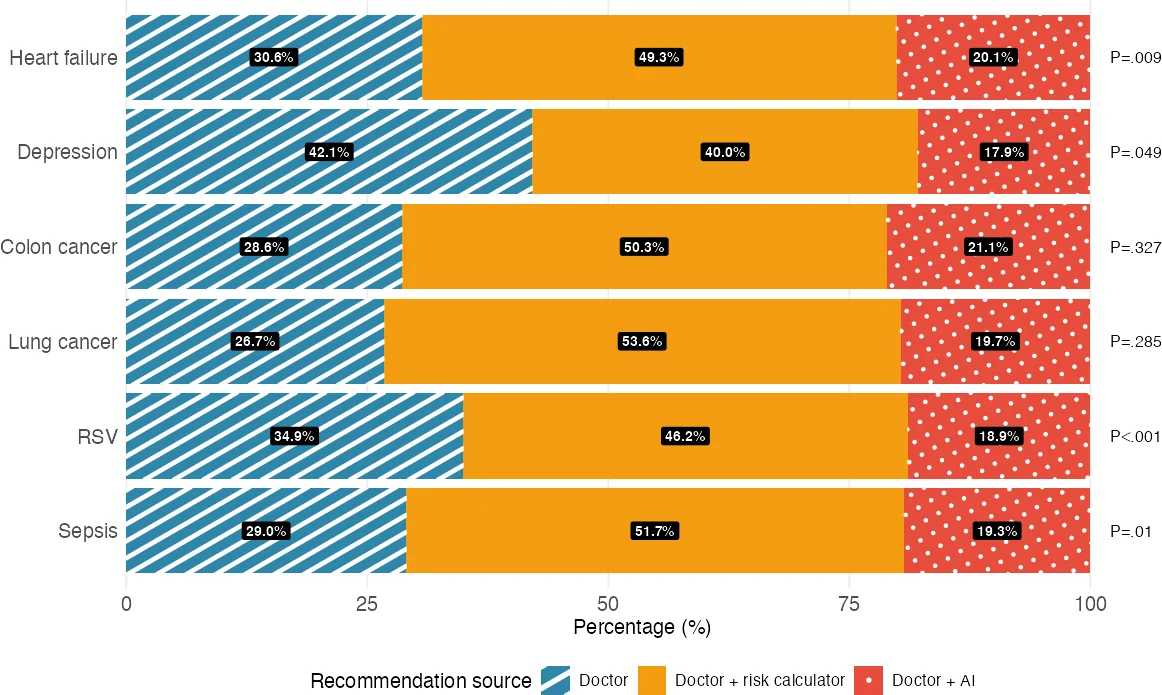
Preference for decision support by clinical condition. RSV: respiratory syncytial virus.

### Qualitative Findings

Of the 1185 participants, open-ended response rates were high across conditions (doctor alone: n=1182, 99.7%; RC: n=1176, 99.2%; AI: n=1172, 98.9%). In open-ended responses, participants highlighted 4 major themes: decision quality and reliability; trust and perceptions; patient-centeredness and autonomy; and fairness, transparency, and ethics. Responses categorized as “lack of concerns/uncertainty” include explicit statements of no concern and blank or nonsubstantive responses. Table 5 provides theme definitions and frequencies by decision-support source.

**Table 5.** Comparison of patient perception qualitative themes across human, risk calculator (RC), and AI.

Theme and subtheme	Description	Human (n=1182), n (%)	RC (n=1176), n (%)	AI (n=1172), n (%)
Decision quality and reliability				
Accuracy and error	Concerns about the correctness of clinical decisions, including potential mistakes or errors.	68 (5.8)	107 (9.1)	100 (8.5)
Overtreatment or misuse of output	Concerns that the decision-maker may recommend unnecessary interventions or misuse decision-support tools.	17 (1.4)	9 (0.8)	9 (0.8)
Capabilities	Concerns about whether the decision-maker (physician, AI, and RC) has the knowledge, expertise, or ability to make appropriate clinical decisions.	116 (9.8)	55 (4.7)	96 (8.2)
Confidence in decision maker	Participants’ expressed confidence in the decision-maker (doctor/AI/RC) within open-ended responses.	95 (8.0)	30 (2.6)	20 (1.7)
Trust and perception				
Trust	General expressions of trust or distrust toward the decision-maker (doctor/AI/RC) within open-ended responses.	22 (1.9)	20 (1.7)	103 (8.8)
Positive perception	Statements reflecting favorable views of the decision-maker (doctor/AI/RC) within open-ended responses.	12 (1.0)	41 (3.5)	38 (3.2)
Negative perception	Statements reflecting skepticism, rejection, or negative attitudes.	16 (1.4)	43 (3.7)	62 (5.3)
Neutral perception	Expressions that are neither clearly positive nor negative.	11 (0.9)	16 (1.4)	14 (1.2)
Fear	Expressions of worry or apprehension regarding the decision maker’s recommendations.	11 (0.9)	6 (0.5)	21 (1.8)
Discomfort	Feelings of unease, uncertainty, or ambivalence.	3 (0.3)	8 (0.7)	25 (2.1)
Patient-centeredness and autonomy				
Joint decision-making	Desire for collaborative decision-making between patient and clinician.	99 (8.4)	67 (5.7)	43 (3.7)
Desire for autonomy/patient inclusion	Explicit statements that patients want to be involved in final decisions or maintain control over their care.	17 (1.4)	5 (0.4)	1 (0.1)
Personalization	Need for recommendations to be tailored to the individual patient’s characteristics, preferences, or needs.	9 (0.8)	37 (3.1)	25 (2.1)
Fairness, transparency, and ethics				
Bias in data and training	Concerns about inequities, discriminatory outcomes, or biased recommendations due to data or algorithm limitations.	17 (1.4)	14 (1.2)	23 (2.0)
Transparency	Desire for clarity in how decisions or recommendations are generated.	7 (0.6)	9 (0.8)	21 (1.8)
Ethics	Broader concerns about ethical implications.	8 (0.7)	5 (0.4)	8 (0.7)
Privacy	Concerns on confidentiality, or privacy in decision-making.	0 (0)	3 (0.3)	6 (0.5)
Lack of concern/uncertainty				
Uninterpretable	Responses that could not be reliably categorized.	155 (13.1)	154 (13.1)	149 (12.7)
No additional concern	Explicit statements of no concern.	478 (40.4)	500 (42.5)	365 (31.1)
Uncertainty of opinion	Expressions of uncertainty or ambivalence that do not map clearly to other themes.	21 (1.8)	47 (4.0)	43 (3.7)

### Decision Quality and Reliability

Participants most frequently raised decision quality and reliability concerns about doctors (n=296, 25%), but also about AI (n=225, 19%) and RCs (n=201, 17%). Doctors were viewed as knowledgeable but fallible. One participant noted, “6 times out of 10 some doctors are right, some are wrong” (P133 on doctors).

Doctors were also sometimes viewed as influenced by external pressures. One participant noted, “They will do what the pharma companies push them to do” (P955 on doctors).

RCs were described as helpful but limited:

*They’re just calculations or sort of like assessments but it’s not a guarantee.*

[P185 on RCs]

*Risk calculators are designed for a single issue and not multiple factors.*

[P587 on RCs]

AI drew skepticism regarding errors and vulnerabilities, with participants providing responses such as:

*The wrong variables might be added.*

[P96 on AI]

*AI makes too many random errors and hallucinations.*

[P448 on AI]

### Trust and Perceptions

Trust and perception concerns were most prominent for AI (n=263, 22%), followed by RCs (n=134, 11%), and least cited for doctors (n=75, 6%). Participants expressed ambivalence across all modalities. Regarding doctors, one participant noted, “not all doctors can be trusted for your health recommendations” (P398 on doctors).

RCs were met with skepticism and negative perceptions. One participant noted, “Its all numbers and everyone is different so I can’t see where this would be helpful” (P474 on RCs).

Opinions on AI varied. Some were optimistic. One participant noted, “AI will change the world of medicine” (P204 on AI).

On the other hand, several comments echoed rejection and mistrust. One participant noted, “I’ll never have faith in a machine!” (P403 on AI).

### Patient-Centeredness and Autonomy

Participants valued patient-centeredness and autonomy across all decision support sources, with these concerns most prominently referenced for doctors (n=125, 11%), followed by RCs (n=109, 9%) and AI (n=69, 6%).

Some participants expressed concern that clinicians may not fully incorporate patient preferences or adequately involve them in decision-making.

Regarding doctors, several participants described concerns about not being sufficiently heard or included:

*Does not take my considerations seriously.*

[P525 on doctors]

*They should discuss it with their patients before making a decision. Sometimes, they are wrong.*

[P8 on doctors]

Others emphasized tensions between clinical recommendations and personal knowledge or preferences:

*That they don’t know what I need or want for my body.*

[P118 on doctors]

*I just feel that sometimes I may know my body better than others.*

[P1074 on doctors]

For RCs, participants again emphasized maintaining autonomy in final decisions. One participant noted, “The risk calculators are an added benefit, but in the end, I want to decide” (P741 on RCs).

AI was more often perceived as insufficiently personalized:

*The AI hasn’t had enough data to make accurate recommendations based on my personal need(s).*

[P525 on AI]

*We are human. It should be treated as a case-by-case scenario. Not everyone is the same.*

[P851 on AI]

### Fairness, Transparency, and Ethics

Concerns about ethics, overtreatment, and bias were raised across sources. Participants cited worries about data bias, privacy, and the transparency of both algorithmic and human decisions.

Some also noted potential hidden agendas and improper data use. Doctors were viewed as susceptible to bias and financial motives. One participant noted, “I worry they are more concerned with what is good for the hospital or their pocket than my health” (P463 on doctors).

RCs raised concerns about overtreatment. One participant noted, “I feel that there may be some unnecessary procedures based on the findings” (P76 on RCs).

AI elicited worries about bias and privacy. One participant noted, “Biases could come into play based on preexisting conditions and the human factor may be ignored and [I] don’t want my information being online” (P726 on AI).

### Lack of Concerns/Uncertainty

A substantial proportion of responses fell into this category, though they reflected distinct underlying sentiments. Most commonly, participants explicitly stated they had no additional concerns (eg, “nothing” and “none at all”), accounting for 40.4% (n=478) of responses for doctors, 42.5% (n=500) for RCs, and 31.1% (n=365) for AI. A smaller proportion expressed uncertainty of opinion (eg, “I don’t know” and “unsure”), reflecting genuine ambivalence rather than an absence of concern (1.8% (n=21) for doctors, 4.0% (n=47) for RCs, and 3.7 % (n=43) for AI). The remainder were uninterpretable responses that were too brief, lacked detail, or did not contain full words or sentences, and, therefore, could not be reliably categorized (for doctors: n=155, 13.1%; for RCs: n=154, 13.1%; for AI: n=149, 12.7%).

## Discussion

### Principal Findings

Our study provides evidence regarding patient perspectives on realistic, yet hypothetical, implementations of AI-informed CDS. Prior work has not examined, within a single comparative study, patients’ reactions to common sources of CDS: a clinician working alone; a clinician using rule-based tools or guidelines (eg, RCs); and a clinician receiving AI support. A 2021 study found that patient participants were more likely to follow recommendations from a doctor alone or a doctor using AI than from an AI system making decisions independently, though this has been one of the few studies to quantitatively compare these decision-support options [21].

In our study, decision source perspectives for behavioral intentions, trust, and preference did not align. Participants reported being likely to take a clinical action (behavioral intention) more often in the doctor + risk score scenarios than in the doctor + AI scenarios. However, participants expressed the greatest trust in doctor-alone scenarios, followed by doctor + RC, and were least trusting of doctor + AI. Yet, similar to behavioral intentions, participants *preferred* the doctor + RC–informed decisions. This divergence between intentions, trust, and preferences may suggest that patients may want clinicians to leverage external, evidence-based tools that can synthesize complex information, yet they remain cautious about tools explicitly labeled as “AI,” although this hypothesis requires further investigation.

We found some consistent and some divergent patterns across behavioral intentions and trust related to the impact of the clinical condition; participants had lower behavioral intentions and trust in the depression (psychiatry referral) and heart failure (go home instead of being admitted) scenarios compared to the sepsis reference scenario. On the other hand, participants had lower behavioral intentions but higher trust for the colon cancer scenarios compared to sepsis. It is important to note that the sepsis scenario involved a proxy decision for a loved one. While it may be challenging to derive conclusive insights on AI use in specific clinical scenarios, our findings do underscore the continued importance of the clinical context and recommended action.

We found differences in both behavioral intentions and trust in certain sociodemographic groups; however, since these analyses were conducted across decision support conditions, they provide more insight into clinical decision-making in general, rather than computer-supported clinical decision-making. Findings reflect what may be anticipated based on prior work related to trust in medical care [38,39]. For example, those who reported experiencing greater discrimination in medical settings also reported lower trust in decision-making regardless of decision support condition. Higher self-rated health literacy and numeracy were also associated with greater trust and behavioral intentions. Future studies may benefit from exploring a single clinical scenario and assessing the interaction between different decision support conditions and sociodemographic characteristics to better understand how participant characteristics may impact perspectives on computer-supported decision support.

Qualitative responses echoed prior literature on reliability, trust, personalization, and bias [40], while adding a side-by-side critique of both computer-supported and human decision-making. Comments revealed interest in transparency in both humans (doctors being influenced by pharmaceutical or other financial incentives) and AI (lack of explanation of results). Participants described doubts and perceived deficiencies for each decision source, reinforcing the idea that all approaches have trade-offs and appropriate use cases. Qualitative findings revealed a potential misconception that AI cannot personalize care. In fact, predictive models can incorporate large volumes of patient-specific data and dynamically adjust variable weights, but this capability is not intuitive to patients. While a substantial share of participants reported no additional concerns (40.4% for clinicians, 42.5% for RCs, and 31.1% for AI), a smaller subset expressed genuine uncertainty of opinion (1.8%, 4.0%, and 3.7%, respectively). This latter group, combined with the potential misconception around AI’s personalization capabilities described above, suggests that gaps in understanding of how AI tools work persist for at least a portion of patients, even as most did not report active concerns.

### Implications for Patient-Centered AI in Clinical Settings

Our findings offer future avenues of investigation for implementing AI in clinically realistic ways. While AI-related safety concerns must be continuously monitored and mitigated, AI can deliver benefits in predictive performance and clinical efficiency. To preserve trust, these benefits should be communicated to patients clearly and transparently.

Qualitative findings highlight the potential value of simplified, inclusive, and digestible general patient-facing education about AI. Many participants wanted to learn how AI works and how it supports their health. Comments about a lack of personalization suggest gaps in understanding AI’s capabilities and limits. Videos and tutorials that explain basic AI concepts may be an engaging starting point; they can be adapted to health care with a strong emphasis on safety and appropriate use. Although the AI decision source did not change participants’ stated likelihood of following recommended clinical actions, we observed lower trust in clinicians supported by AI for predictive tasks and stronger preferences for rule-based “risk calculators” with easily explainable outputs. Given ethical obligations and legal initiatives [6,7], it is important to disclose when AI supports treatment recommendations. To avoid eroding trust, disclosures should provide context-specific information about the tools used. This can include information such as how and when the AI adds value beyond human judgment, performance on representative populations, data provenance and uncertainty, and assurances that clinicians remain in control. In alignment with prior work, information should make system capabilities and limitations visible and support efficient correction and oversight [41]. These and previous results suggest that information should also be tailored to the clinical condition, as people value actionable, question-driven transparency over generic disclosures [42]. Implementation may benefit from reusable design patterns and templates that can be customized to the site and clinical context. Providing Fast Healthcare Interoperability Resources–based components and detailed implementation guides may support scalability across settings. Participants appeared more receptive to terms like “risk calculator” than “AI.” They also valued explainability, underscoring the importance of patient-facing explanations (eg, salient features or illustrative examples). This work should draw on decades of research in patient risk communication to avoid reinventing the wheel [43,44]. All of these approaches should be coupled with rigorous human-centered design evaluations that can assess the efficacy of these approaches, which we did not directly evaluate in our study.

### Limitations and Future Work

Our study has several limitations that also provide implications for future work. Our quantitative findings were likely influenced by our provided definitions of RCs and AI. We had to generate our own patient-facing descriptions, as health care–centric, lay definitions of AI and related topics were not found on sites such as MedlinePlus. The informatics, risk communication, and human-computer interaction communities may support generating and coming to consensus on standardized lay-focused definitions for key AI vocabulary to mitigate this issue in the future. Given the nature of our approach, we were not able to conduct investigations regarding sociodemographic differences with the qualitative data. Future qualitative investigations may use maximum variance sampling to better tease apart potential differences.

Our survey involved sampling from an opt-in online panel, hypothetical clinical decisions, and stated behavioral intentions rather than actual clinical behavior. Future work should consider enrolling patients experiencing specific clinical conditions and measuring how different implementations of AI tools affect actual health behaviors.

Our study was also conducted only with adults in the United States. We were also unable to account for previous experiences with particular clinical conditions, which may have influenced the trust and behavioral intentions of individual respondents. Moreover, as the scenarios involved hypothetical medical decisions rather than actual care decisions, the participants’ responses could differ when compared with a real-world setting. Future work should assess similar research questions in other settings and may also benefit from understanding how patients’ lived experiences concretely affect their perspectives.

We also did not formally test for order or carryover effects across the six scenarios; while our design prevented consecutive repetition of the same recommendation source, future work could examine whether scenario position influenced responses.

### Conclusions

We found that participants had greater behavioral intentions to take action and preferred scenarios where clinicians were supported by explainable, rule-based tools, such as RCs. Clinicians, however, remained the most trusted source for treatment recommendations, yet many participants preferred doctors using RCs—suggesting a desire for tool-supported judgment without ceding authority to AI. The nature of clinical conditions and actions also affected behavioral intentions and trust. The heterogeneity we observed, including condition-specific and sociodemographic differences, argues for context-aware deployment and co-design with diverse communities to avoid widening disparities. Together, these findings support a pragmatic path forward: use AI where it clearly improves access and efficiency, keep clinicians in the loop for consequential decisions—especially in mental health—and pair implementation with patient education, rigorous evaluation, and equitable deployment.

## Supplementary material

10.2196/100388 Multimedia Appendix 1 Full survey.

10.2196/100388 Multimedia Appendix 2 Full regression data.

10.2196/100388 Multimedia Appendix 3 Additional numerical data from figures and subgroup sample sizes.

10.2196/100388 Multimedia Appendix 4 Claude transcripts.

10.2196/100388 Checklist 1 CHERRIES checklist.

## Abbreviations

CDS: clinical decision support
CHERRIES: Checklist for Reporting Results of Internet E-Surveys
Gen X: Generation X
Gen Z: Generation Z
OR: odds ratio
RC: risk calculator
RSV: respiratory syncytial virus
