# Supplementary material for: Investigating Patient Trust and Intention to Act in AI-Supported Clinical Decisions: Cross-Sectional Survey
Source: J Med Internet Res. 2026 Nov 2;28:e100388. doi: 10.2196/100388 (PMC13741335; doi:10.2196/100388)
Supplement: Supplementary file 1 [file jmir-v28-e100388-s001.pdf]

Start of Block: Age

*

Birth_year In what year were you born? Please enter as a 4-digit number (e.g., 1990, 2001).

____

Display this question:

If If In what year were you born? Please enter as a 4-digit number (e.g., 1990, 2001). Text Response Is Equal to 2006

Q327 Are you currently at least 18 years old?

- ◯ Yes
- ◯ No

End of Block: Age

Start of Block: Consent Form

consent_instructions **Please review the information sheet linked below.**

consent_yn By clicking “yes” you are indicating that you voluntarily agree to participate in this study. Note: if you click "no" the survey will end now.

- ◯ Yes, I consent.
- ◯ No, I do not consent (survey will end).

End of Block: Consent Form

Start of Block: Demographics

sex_at_birth What sex were you assigned at birth, on your original birth certificate?

- ◯ Male
- ◯ Female
- ◯ Don't Know
- ◯ Prefer not to answer

---

gender What is your current gender?

- ◯ Woman
- ◯ Man
- ◯ Transgender
- ◯ Nonbinary
- ◯ Don't Know
- ◯ Prefer Not To Answer

---

race_select_multiple What race(s) do you identify as?

- ☐ American Indian or Alaska Native
- ☐ Asian
- ☐ Black or African American
- ☐ Native Hawaiian or Other Pacific Islander
- ☐ White
- ☐ Prefer Not to Answer

Display this question:

If If What race(s) do you identify as? q://QID1318852866/SelectedChoicesCount Is Greater Than 1

Carry Forward Selected Choices from "What race(s) do you identify as? "

X→

race_select_one With which race do you most identify?

- ○ American Indian or Alaska Native
- ○ Asian
- ○ Black or African American
- ○ Native Hawaiian or Other Pacific Islander
- ○ White
- ○ Prefer Not to Answer

---

ethnicity What ethnicity do you identify as?

- ◯ Hispanic/Latino
- ◯ Not Hispanic/Latino
- ◯ Unsure/prefer not to answer

---

coutry_of_origin What is your country of origin (e.g., United States, Mexico, Canada)?

____

---

financial_stability Do you feel you have enough financial resources to make ends meet?

- ◯ More than enough
- ◯ Enough
- ◯ Not enough
- ◯ Unsure/Prefer Not to Answer

---

education What is the highest level of education you completed?

- ○ Did not complete high school or receive GED
- ○ High school diploma or GED
- ○ Some college or Associates degree (for example: AA, AS)
- ○ Bachelor's degree (for example: BA. BS)
- ○ Master's degree (for example: MA, MS, MEng, MEd, MSW, MBA)
- ○ Professional degree beyond bachelor's degree (for example: MD, DDS, DVM, LLB, JD)
- ○ Doctorate degree (for example, PhD, EdD)
- ○ Prefer Not to Answer

---

community_residence Which of the following best describes the community in which you reside?

- ○ Rural
- ○ Suburban
- ○ Urban
- ○ Other ____
- ○ Don't Know/Prefer Not to Answer

---

insurance_coverage Have you had any type of health insurance coverage in the last year?

- ◯ Yes, for the full year
- ◯ Yes, for part of the year
- ◯ No
- ◯ Prefer Not to Answer

Display this question:

If Have you had any type of health insurance coverage in the last year? = Yes, for the full year

Or Have you had any type of health insurance coverage in the last year? = Yes, for part of the year

insurance_type If yes, have you been covered be any of the following types of health insurance of health coverage plans in the past year?

- ▢ Insurance through a current or former employer or union (of this person or another family member)
- ▢ Insurance purchased directly from an insurance company (by this person or another family member)
- ▢ Medicaid, Medical Assistance, or any kind of government-assistance plan for those with low incomes or a disability
- ▢ TRICARE or other military health care
- ▢ VA (enrolled for VA health care)
- ▢ Another type of health insurance or health coverage plan

____

---

healthprofessional Are you a health professional? If yes, please specify your role as a health professional (e.g., physician, nurse).

- ◯ Yes ____
- ◯ No
- ◯ Not Sure

End of Block: Demographics

Start of Block: Instructions

instructions Doctors can use different tools along with their own knowledge to make health decisions. We want to understand what tools, if any, you would feel comfortable with your doctor using to make healthcare decisions. Specifically, we want to know your thoughts about "risk calculators" and "artificial intelligence" . These tools are described in more detail below.

Risk calculators allow doctors, nurses, and patients to enter information about a patient (such as, age, gender, smoking status) and calculate a number that shows a patient's risk, for example, of having a stroke (see picture below). **These tools can easily explain how they get to the risk prediction because they use simple math.**

Artificial intelligence (AI) or "AI", refers to computer programs that can learn to make decisions or predictions like humans using large amounts of data. AI, for example, may take large amounts of data from patients they know did and did not have a stroke in their lifetime. The AI learns patterns from this data and predicts who is at high risk of stroke in the future. **AI is different from a risk calculator in that it cannot always explain the rules it used to make a prediction.**

---

inst_comprehension From these descriptions, what is the main difference between risk calculators and AI?

- ◯ AI is more accurate than risk calculators
- ◯ Risk calculators can easily explain the risk prediction, AI cannot always do this.
- ◯ Risk calculators use rules that are hard to explain
- ◯ AI uses less information than risk calculators

Display this question:

If From these descriptions, what is the main difference between risk calculators and AI? = Risk calculators can easily explain the risk prediction, AI cannot always do this.

feedback_right Yes. The main difference between AI and risk calculators to remember is that risk calculators can easily explain the risk prediction but AI cannot always do this.

Display this question:

If From these descriptions, what is the main difference between risk calculators and AI? != Risk calculators can easily explain the risk prediction, AI cannot always do this.

feedback_wrong Not quite. The main difference between AI and risk calculators to remember is that risk calculators can easily explain the risk prediction but AI cannot always do this.

Q325 The next questions in this survey will ask about your thoughts on how risk calculators or AI may be used to help make decisions for 6 different medical situations. The questions will be similar for each situation. The survey will finish with a few more questions about AI and some questions about yourself.

End of Block: Instructions

Start of Block: Lung Cancer (LC) 1_D+AI

****Only one block of questions included as an example, not all counterbalanced conditions****

LC_background_dai **Lung cancer** is cancer that forms in tissues of the lung. Lung cancer is typically first seen on a chest x-ray and confirmed through a test called a "**biopsy**". **Biopsy use and benefits:** A biopsy removes a small amount of cells or tissue from your body. A doctor looks at the cells or tissue under a microscope to check for damage or disease, such as cancers. A needle biopsy removes tissue with a needle passed through your skin to the site of the problem. Other kinds of biopsies may require surgery. **Biopsy side effects:** During a biopsy, the care team will give pain medication so you do not feel the needle being inserted. After a biopsy, you may experience some soreness at the biopsy site or cough up a little blood, but this should be minimal. These symptoms will gradually fade over the 12 to 48 hours following the procedure.

LC_familar_dai How familiar are you with lung cancer screening or treatment?

- ◯ Not familiar at all
- ◯ Slightly familiar
- ◯ Somewhat familiar
- ◯ Very familiar

Page Break

LC_scenario_dai **Scenario:** You are 55 years old and have had a sinus infection with a cough for several weeks. Your doctor decides to order a chest x-ray to check for pneumonia. After the x-ray, **your doctor tells you that with the help of a AI,** a spot on your x-ray suspicious for lung cancer was identified. It is recommended you get a biopsy next week.   Please answer the following questions based on the scenario above:

LC_affriskdis_dai How worried are you about having lung cancer?

- ○ Not at all worried
- ○ Slightly worried
- ○ Neutral
- ○ Moderately worried
- ○ Very worried

LC_affrisktreat_dai How worried are you about having the biopsy and its side effects?

- ○ Not at all worried
- ○ Slightly worried
- ○ Neutral
- ○ Moderately worried
- ○ Very worried

LC_behavintent_dai How likely are you to get a biopsy?

- ◯ Extremely unlikely
- ◯ Somewhat unlikely
- ◯ Neutral
- ◯ Somewhat likely
- ◯ Extremely likely

LC_trust_dai To what extent do you agree with this statement: I trust the **doctor and AI** to make a recommendation about a biopsy for lung cancer.

- ◯ Not at all
- ◯ A little
- ◯ Neutral
- ◯ Somewhat
- ◯ Very much

End of Block: Lung Cancer (LC) 1_D+AI

Start of Block: Colon Cancer (CC) 1_D+R

CC_background_drc **Colon cancer** (or colorectal cancer) is cancer that develops in the tissues of the colon or rectum, which are part of your digestive system. A **colonoscopy** is a procedure used to identify health issues within your colon. **Colonoscopy uses and benefits:** Colonoscopy is a procedure that lets your doctor look inside your rectum and colon (large intestine). They use instruments called scopes with a light and a tiny camera attached to a long, thin tube that let your doctor see problems such as cancer. **Colonoscopy - possible drawbacks:** To prepare for a colonoscopy, you will need to follow a clear liquid diet, usually for about one day before the procedure. You probably need to stop eating and drinking the night before the exam. You will need to take some laxatives that will cause diarrhea to clear out your colon and rectum.

CC_familar_drc How familiar are you with colon cancer screening or treatment?

- Not familiar at all
- Slightly familiar
- Somewhat familiar
- Very familiar

Page Break

CC_scenario_drc **Scenario:** You are 40 years old and moderately healthy. During your annual physical exam, **your doctor informs you that with the help of a risk calculator** they determined that you are at higher-than-average risk for colon cancer. They recommend you get a colonoscopy in the next month. Please answer the following questions based on the scenario above:

CC_affriskdis_drc How worried are you about having colon cancer?

- ○ Not at all worried
- ○ Slightly worried
- ○ Neutral
- ○ Moderately worried
- ○ Very worried

CC_affrisktreat_drc How worried are you about having the colonoscopy and its side effects?

- ○ Not at all worried
- ○ Slightly worried
- ○ Neutral
- ○ Moderately worried
- ○ Very worried

CC_behavintent_drc How likely are you to get a colonoscopy?

- ○ Extremely unlikely
- ○ Somewhat unlikely
- ○ Neutral
- ○ Somewhat likely
- ○ Extremely likely

CC_trust_drc To what extent do you agree with this statement: I trust the **doctor and risk calculator** to make a recommendation about a colonoscopy for colon cancer.

- ○ Not at all
- ○ A little
- ○ Neutral
- ○ Somewhat
- ○ Very much

End of Block: Colon Cancer (CC) 1_D+R

Start of Block: Depression (Dep) 1_D

Dep_background_d **Depression** is a serious medical illness. It's more than just a feeling of being sad or "blue" for a few days. These feelings persist and interfere with your everyday life. Depression is often treated by a psychiatrist. **Psychiatrist use and benefits:** A psychiatrist is a medical doctor (an M.D. or D.O.) who specializes in mental health. Psychiatrists use a variety of treatments including talk therapy, medications, psychosocial interventions and other treatments (such as electroconvulsive therapy or ECT), depending on the needs of each patient. **Psychiatrist - possible drawbacks:** Seeing a psychiatrist usually involves a conversation with them about how you are feeling mentally and may cover sensitive topics. Psychiatrists may not take insurance, so you may also have out of pocket costs (not covered by insurance).

Dep__familar_d How familiar are you with depression screening or treatment?

- ◯ Not familiar at all
- ◯ Slightly familiar
- ◯ Somewhat familiar
- ◯ Very familiar

Page Break

Dep_scenario_d **Scenario:** You are 30 years old and go to your yearly visit with your primary care provider. After the exam and discussion, the **doctor** informs you, they determined that you are at a high-risk for developing depression in the next year. Your doctor provides you a referral to a psychiatrist (doctor that specializes in mental health). Please answer the following questions based on the scenario above:

Dep_affriskdis_d How worried are you about having depression?

- ○ Not at all worried
- ○ Slightly worried
- ○ Neutral
- ○ Moderately worried
- ○ Very worried

Dep_affrisktreat_d How worried are you about the drawbacks of seeing a psychiatrist?

- ○ Not at all worried
- ○ Slightly worried
- ○ Neutral
- ○ Moderately worried
- ○ Very worried

Dep_behavintent_d How likely are you to see a psychiatrist?

◯ Extremely unlikely

◯ Somewhat unlikely

◯ Neutral

◯ Somewhat likely

◯ Extremely likely

Dep_trust_d To what extent do you agree with this statement: I trust the doctor to make a recommendation about seeing a psychiatrist for depression.

◯ Not at all

◯ A little

◯ Neutral

◯ Somewhat

◯ Very much

End of Block: Depression (Dep) 1_D

Start of Block: Attention Check 1

Q1 The test you are about to take part in is very simple: When asked how important eating breakfast is you must select "Very important". This is an attention check.

◯ Not Important

◯ Somewhat Important

◯ Very Important

◯ Don't Know

End of Block: Attention Check 1

Start of Block: Heart Failure (HF) 1_D+AI

HF_background_dai **Heart failure** means that your heart can't pump enough oxygen-rich blood to meet your body's needs, which causes fluid to build up in the body. When too much fluid builds up in the body patients have swelling and shortness of breath that results in an emergency room visit. It is typical for patients to be admitted to the hospital (hospitalized) to receive medications to help get the fluid out of the body.   **Benefits of sending low-risk heart failure patients home:** Often patients are hospitalized even though they have a low risk of heart failure problems. By being sent home when they are at low-risk patients can reduce care costs. Hospitals can also make sure there are enough resources in the hospital for patients who need them most. Also, patients can avoid the negative effects of being hospitalized such as a higher risk of falling and hospital-acquired infections   **Challenges of sending low-risk heart failure patients home:** Patients who are released home may have their symptoms get worse, and they will have to go back to the hospital.

HF_familar_dai How familiar are you with heart failure and its treatment?

- ○ Not familiar at all
- ○ Slightly familiar
- ○ Somewhat familiar
- ○ Very familiar

Page Break

HF_scenario__dai **Scenario:** You are 65 years old and were diagnosed with heart failure 10 years ago. Several times each year you become short of breath, tired, and nauseous, and you go to the Emergency Room (ER), where they admit you for a brief hospital stay. This time, when you are in the ER for the same symptoms, **the doctor with the help of an AI-based algorithm**, determines that you have a low risk for serious complications in the next 30 days and recommends that you go home and manage the fluid with medications yourself. Please answer the following questions based on the scenario above:

HF_affriskdis_dai How worried are you about being admitted to a hospital for your heart failure?

- ○ Not at all worried
- ○ Slightly worried
- ○ Neutral
- ○ Moderately worried
- ○ Very worried

HF_affrisktreat_dai How worried are you about having a bad outcome if you go home instead of being admitted?

- ○ Not at all worried
- ○ Slightly worried
- ○ Neutral
- ○ Moderately worried
- ○ Very worried

HF_behavintent_dai How likely are you to agree to go home instead of being admitted?

- Extremely unlikely
- Somewhat unlikely
- Neutral
- Somewhat likely
- Extremely likely

HF_trust_dai To what extent do you agree with this statement: I trust the **doctor and AI** to make a recommendation about whether to go home instead of being admitted for my heart failure.

- Not at all
- A little
- Neutral
- Somewhat
- Very much

End of Block: Heart Failure (HF) 1_D+AI

Start of Block: Sepsis (Sep) 1_D+R

Sep_background_drc **Sepsis** is the body's overactive and extreme response to an infection. Sepsis can be hard to diagnose because symptoms can imitate a lot of other health problems. Sepsis is a life-threatening medical emergency so it is very important to get treatment right away. Treatment usually includes antibiotics and maintaining blood flow to organs. This may involve getting oxygen and high volume intravenous (IV) fluids. **Benefits of treating sepsis with high-volume IV fluids:** High-volume IV fluids provide hydration and help flush infection from a patients body. They can be helpful for infections, such as sepsis. **Risks of treating sepsis with high-volume IV fluids**: Sepsis patients with heart failure may have a harder time than most getting rid of the extra IV fluids given during treatment which can cause more heart problems. Therefore, it is important to be very sure that patients with heart failure need extra IV fluids before they get them.

Sep_familar_drc How familiar are you with sepsis and its treatment?

- ○ Not familiar at all
- ○ Slightly familiar
- ○ Somewhat familiar
- ○ Very familiar

Page Break

Sep_scenario_drc **Scenario:** Your 80-year old father with heart failure is in the Intensive Care Unit following a planned surgery, and the doctors say he is recovering well. The attending **doctor informs you that with the help of a risk calculator**, they determined that your father is at high risk for developing sepsis, even though he seems ok now, and they recommend starting antibiotics and a high-volume IV. Please answer the following questions based on the scenario above:

Sep_affriskdis_drc How worried are your father becoming septic (developing sepsis)?

- ◯ Not at all worried
- ◯ Slightly worried
- ◯ Neutral
- ◯ Moderately worried
- ◯ Very worried

Sep_affrisktreat_drc How worried are you about the side effects your father may have from sepsis treatments?

- ◯ Not at all worried
- ◯ Slightly worried
- ◯ Neutral
- ◯ Moderately worried
- ◯ Very worried

Sep_behavintent_drc How likely are you agree to having them treat your father for sepsis?

- Extremely unlikely
- Somewhat unlikely
- Neutral
- Somewhat likely
- Extremely likely

Sep_trust_drc To what extent do you agree with this statement: I trust the doctor and risk calculator to make a recommendation about starting treatment for sepsis.

- Not at all
- A little
- Neutral
- Somewhat
- Very much

End of Block: Sepsis (Sep) 1_D+R

Start of Block: RSV Vaccination (RSV) 1_D

RSV_background_d **Respiratory syncytial virus**, or **RSV**, is a common respiratory virus. It usually causes mild, cold-like symptoms. But it can cause serious lung infections, especially in infants, older adults, and people with serious medical problems. The RSV vaccine can prevent lower respiratory tract disease. **RSV vaccine uses and benefits:** RSV vaccine can prevent lower respiratory tract disease caused by respiratory syncytial virus (RSV).   **RSV vaccine side effects and drawbacks:** Side effects of the RSV vaccine can include pain, redness, and swelling where the shot is given, fatigue (feeling tired), fever, headache, nausea, diarrhea, and muscle or joint pain. These are usually temporary and go away within a few days.

RSV_familar_d How familiar are you with RSV and its prevention?

- ○ Not familiar at all
- ○ Slightly familiar
- ○ Somewhat familiar
- ○ Very familiar

RSV_safe_d In general, how safe would you say vaccines are?

- ○ Not at all safe
- ○ A little safe
- ○ Moderately safe
- ○ Very safe

Page Break

RSV_scenario_d **Scenario:** You are 70-years old and seeing your primary care provider for a routine annual visit in the fall. **Your doctor** informs you that you are at a high-risk for getting and having a severe case of RSV based on your age and the level of infections in your area, and they recommend that you get the RSV vaccine. Please answer the following questions based on the scenario above:

RSV_affriskdisease_d How worried are you about getting RSV?

- ○ Not at all worried
- ○ Slightly worried
- ○ Neutral
- ○ Moderately worried
- ○ Very worried

RSV_affrisktreat_d How worried are you about the side effects you may have from the RSV vaccine treatments?

- ○ Not at all worried
- ○ Slightly worried
- ○ Neutral
- ○ Moderately worried
- ○ Very worried

RSV_behavintent_d How likely are you agree to having the RSV vaccine?

- Extremely unlikely
- Somewhat unlikely
- Neutral
- Somewhat likely
- Extremely likely

RSV_trust_d To what extent do you agree with this statement: I trust the **doctor** to make a recommendation about my risk of getting RSV.

- Not at all
- A little
- Neutral
- Somewhat
- Very much

End of Block: RSV Vaccination (RSV) 1_D

Start of Block: Preference & Concerns

Q343 For each medical situation you reviewed, please choose your preference for how medical recommendations are made.

---

pref_LC Whether or not to receive a biopsy for **lung cancer**.

- Doctor/health professional decides alone
- Doctor/health professional decides with the help of a risk calculator
- Doctor/health professional decides with the help of AI

---

pref_C Whether or not to recommend a colonoscopy for possible **colon cancer**.

- Doctor/health professional decides alone
- Doctor/health professional decides with the help of a risk calculator
- Doctor/health professional decides with the help of AI

---

pref_HF Whether or not to send home someone with a low risk of **heart failure** complications.

- Doctor/health professional decides alone
- Doctor/health professional decides with the help of a risk calculator
- Doctor/health professional decides with the help of AI

---

pref_Dep Whether or not to see a psychiatrist for possible **depression**.

- Doctor/health professional decides alone
- Doctor/health professional decides with the help of a risk calculator
- Doctor/health professional decides with the help of AI

---

pref_Sep Whether or not to begin **sepsis** treatment for your father with heart failure.

- ◯ Doctor/health professional decides alone
- ◯ Doctor/health professional decides with the help of a risk calculator
- ◯ Doctor/health professional decides with the help of AI

---

pref_RSV Whether or not to receive a vaccine to protect against **RSV** (respiratory synthetic virus).

- ◯ Doctor/health professional decides alone
- ◯ Doctor/health professional decides with the help of a risk calculator
- ◯ Doctor/health professional decides with the help of AI

---

open_doctor Please write any concerns you have about doctors alone making recommendations for your health care.

____

____

____

____

____

---

open_RiskCalc Please write any concerns you have about risk calculators being used to inform your health care.

____

____

____

open_AI Please write any concerns you have about AI being used to inform your health care.

____

____

____

____

____

End of Block: Preference & Concerns

Start of Block: GenAI

Intro_GenAI There is another kind of AI that learns from large amounts of text or word-based data. This AI, known as **generative AI**, can be used to create text, like a summary of a patient visit, or answer questions, for example, about common symptoms for a certain disease. An example of generative AI that you may have heard of is called ChatGPT.

Page Break

Patient_portal_scen **Scenario: Using Generative AI for Patient Portal Secure Messaging** Assume, generative AI specific to medical information has been created at your health system and could be used to reliably answer messages you send to your doctor, such as in a patient portal. For each kind of message you might send, would you prefer to receive a fast answer from the AI (within 5 minutes) **OR** wait until a health professional (doctor or nurse) may review and edit the AI's answer (up to 48 hours)?

---

portal_general ***General information messages***: such as asking for general information about diseases, symptoms, treatment strategies, or other medical topics.

○ Faster AI Answer

○ Slower Doctor approved answer

---

portal_medical ***Medical messages:*** specific to your own health, such as request for or information about: following up on a referral or prescribed therapy, reporting new symptoms or problems, or test result interpretation.

○ Faster AI Answer

○ Slower Doctor approved answer

---

portal_logistical ***Logistical messages:*** questions or requests about the office's contact information, policies (e.g., appointment cancellation, availability of services), insurance/billing, or requesting personal information/medical records.

○ Faster AI Answer

○ Slower Doctor approved answer

---

patient_portal-free Please write any reasons you may have for the answers you gave to the last three questions.

____

Page Break

Chatbot Therapy **Scenario: Using Generative AI for Mental Health Support** Lately, you have been feeling both down and anxious about your future. You reach out to your primary care provider to understand what kind of support may be available that does not involve medications, such as talking to a therapist. They give you the following options. A talk therapy program with a licensed therapist. Their next available slot to begin is in three months. A generative AI "conversational agent" program (computer program developed from conversations with real therapists) that is monitored by a licensed therapist. The next available slot to start this program is one month away. A generative AI "conversational agent" on its own. You can start using this program immediately, and it is available whenever you need it.

---

chatbot_ther How likely would you be to use the following treatment option: A talk therapy program with a licensed therapist

- Extremely unlikely
- Somewhat unlikely
- Neither likely nor unlikely
- Somewhat likely
- Extremely likely

---

chatbot_chat+ther_BI How likely would you be to use the following treatment option: A conversational agent program that is monitored by a licensed therapist.

- Extremely unlikely
- Somewhat unlikely
- Neither likely nor unlikely
- Somewhat likely
- Extremely likely

---

chatbot_chat_BI How likely would you be to use the following treatment option: A conversational agent

○ Extremely unlikely

○ Somewhat unlikely

○ Neither likely nor unlikely

○ Somewhat likely

○ Extremely likely

Chatbot-free Please write any reasons you may have for the answers you gave to the last three questions.

____

____

____

____

____

End of Block: GenAI

Start of Block: Attention Check 2

Q328 The rest of the questions ask about yourself and experience with healthcare.

Q342 Which age generation are you a member of?

- Baby Boomer (born between 1946 and 1964)
- Gen X (born between 1965 and 1980)
- Millennial (born between 1981 and 1996)
- Gen Z (born between 1997 and 2012)

End of Block: Attention Check 2

Start of Block: Control Preferences Scale

control_pref_scale In terms of making decisions about your health care with your doctor, which ONE of the following best describes how you would like to make these decisions?

- Make the final selection about which treatment I will receive
- Make the final selection after seriously considering my doctor's opinion
- Have my doctor and I share responsibility for deciding what treatment is best
- Have my doctor make the final decision but consider my opinion
- Leave all decisions regarding treatment to my doctor
- Unsure/Prefer Not to Answer

End of Block: Control Preferences Scale

Start of Block: Health Literacy (Chew)

healthlit_1 How often do you have problems learning about your medical condition because of difficulty reading hospital materials?

- None of the time
- A little of the time
- Some of the time
- Most of the time
- All of the time
- Unsure/Prefer Not to Answer

healthlit_2 How confident are you filling out forms by yourself?

- Extremely
- Quite a bit
- Somewhat
- A little bit
- Not at all
- Unsure/Prefer Not to Answer

healthlit_3 How often do you have someone help you read hospital materials?

- None of the time
- A little of the time
- Some of the time
- Most of the time
- All of the time
- Unsure/Prefer Not to Answer

End of Block: Health Literacy (Chew)

Start of Block: Numeracy (SNS)

numeracy_1 How good are you at working with fractions?

- Not good at all
- Slightly good
- Somewhat good
- Moderately good
- Very good
- Extremely good
- Unsure/Prefer Not to Answer

numeracy_2 How good are you at figuring out how much a shirt will cost if it is 25% off?

- ○ Not good at all
- ○ Slightly good
- ○ Somewhat good
- ○ Moderately good
- ○ Very good
- ○ Extremely good
- ○ Unsure/Prefer Not to Answer

---

numeracy_3 How often do you find numerical information to be useful?

- ○ Never
- ○ Very rarely
- ○ Rarely
- ○ Sometimes
- ○ Often
- ○ Very Often
- ○ Unsure/Prefer Not to Answer

End of Block: Numeracy (SNS)

Start of Block: Technology Comfort

tech_comfort How comfortable are you with new technology?

- I am among the first of my peers to adopt the latest technology
- I like to see how new technology works for others before I adopt it
- I tend to wait until the technology is well established before I adopt it
- I reluctantly adopt new technology

End of Block: Technology Comfort

Start of Block: Discrimination in Medical Settings (DMS7)

Q333 Think of your prior experiences while getting health care when answering these questions.

DMS7_1 You are treated with less courtesy than other people.

- Never
- Rarely
- Sometimes
- Most of the time
- Always
- Unsure/Prefer not to answer

DMS7_2 You are treated with less respect than other people.

- Never
- Rarely
- Sometimes
- Most of the time
- Always
- Unsure/Prefer not to answer

DMS7_3 You receive poorer service than others.

- Never
- Rarely
- Sometimes
- Most of the time
- Always
- Unsure/Prefer not to answer

DMS7_4 A doctor or nurse acts as if he or she thinks you are not smart.

- Never
- Rarely
- Sometimes
- Most of the time
- Always
- Unsure/Prefer not to answer

DMS7_5 A doctor or nurse acts as if he or she is afraid of you.

- Never
- Rarely
- Sometimes
- Most of the time
- Always
- Unsure/Prefer not to answer

DMS7_6 A doctor or nurse acts as if he or she is better than you.

- Never
- Rarely
- Sometimes
- Most of the time
- Always
- Unsure/Prefer not to answer

DMS7_7 You feel like a doctor or nurse is not listening to what you were saying

- Never
- Rarely
- Sometimes
- Most of the time
- Always
- Unsure/Prefer not to answer

End of Block: Discrimination in Medical Settings (DMS7)
