# Supplementary material for: Investigating Patient Trust and Intention to Act in AI-Supported Clinical Decisions: Cross-Sectional Survey
Source: J Med Internet Res. 2026 Nov 2;28:e100388. doi: 10.2196/100388 (PMC13741335; doi:10.2196/100388)
Supplement: Supplementary file 2 [file jmir-v28-e100388-s005.pdf]

Checklist for Reporting Results of Internet E-Surveys (CHERRIES)

Checklist Item	*Explanation*	*Details for the Present Study*
Describe survey design	Describe target population, sample frame. Is the sample a convenience sample? (In “open” surveys this is most likely.)	See the subsection “Study Design and Participant Recruitment”<br><br>We recruited participants via Qualtrics’ survey sampling service.<br><br>Eligibility criteria included being at least 18 years old, being able to read English, and providing informed consent within the Qualtrics platform before beginning the survey.<br><br>To support comparisons across sociodemographic groups, we used quota sampling balanced on sex, education (with vs. without a bachelor’s degree or higher), and generation (Baby Boomer, Gen X, Millennial, Gen Z). We also targeted approximately half of the sample to identify as members of racial or ethnic minority groups.
IRB approval	Mention whether the study has been approved by an IRB.	See the subsection “Study Design and Participant Recruitment”<br><br>The Columbia University Institutional Review Board approved this study.
Informed consent	Describe the informed consent process. Where were the participants told the length of time of the survey, which data were stored and where and for how long, who the investigator was, and the purpose of the study?	See Multimedia Appendix 2 (the full survey).<br><br>Informed consent was checked at the beginning of the survey. The information sheet provided contained the length of the survey (15 minutes), as well as the

		purpose of the study, and information on the principal investigator.
Data protection	If any personal information was collected or stored, describe what mechanisms were used to protect unauthorized access.	See the subsection "Study Design and Participant Recruitment"<br><br>No personal information that would allow identification of participants was collected during the survey.
Development and testing	State how the survey was developed, including whether the usability and technical functionality of the electronic questionnaire had been tested before fielding the questionnaire.	See the subsection "Survey Development".<br><br>We developed survey items with input from experts in human factors, anthropology, human-centered AI, AI development, nursing, primary care, and survey design. Our team iteratively drafted and refined the items through multiple rounds of pilot testing with both experts and laypersons to assess comprehensibility and face validity.
Open survey versus closed survey	An "open survey" is a survey open for each visitor of a site, while a closed survey is only open to a sample which the investigator knows (password-protected survey).	See the subsection "Study Design and Participant Recruitment".<br><br>Closed survey.
Contact mode	Indicate whether or not the initial contact with the potential participants was made on the Internet. (Investigators may also send out questionnaires by mail and allow for Web-based data entry.)	See the subsection "Study Design and Participant Recruitment".<br><br>Initial contact was made on the Internet (via Qualtrics).
Advertising the survey	How/where was the survey announced or advertised? Some examples are offline media (newspapers), or online (mailing lists – If yes, which ones?) or banner ads (Where were these banner ads posted and what did they look like?). It is important to know the wording of the announcement as it will heavily influence who chooses to participate. Ideally the survey announcement should be published as an appendix.	See the subsection "Study Design and Participant Recruitment".<br><br>We recruited participants via Qualtrics' survey sampling service, which distributes the survey to its panel respondents.

Web/E-mail	State the type of e-survey (eg, one posted on a Web site, or one sent out through e-mail). If it is an e-mail survey, were the responses entered manually into a database, or was there an automatic method for capturing responses?	See the subsection “Study Design and Participant Recruitment”.<br><br>The survey was managed and distributed through Qualtrics, which manages distributing the survey and collecting the results as participants complete the survey.
Context	Describe the Web site (for mailing list/newsgroup) in which the survey was posted. What is the Web site about, who is visiting it, what are visitors normally looking for? Discuss to what degree the content of the Web site could pre-select the sample or influence the results. For example, a survey about vaccination on a anti-immunization Web site will have different results from a Web survey conducted on a government Web site	See the subsection “Study Design and Participant Recruitment”.<br><br>Qualtrics is widely used for survey research, and its sampling services provides access to a broad audience of participants who are individuals that complete surveys for compensation.
Mandatory/voluntary	Was it a mandatory survey to be filled in by every visitor who wanted to enter the Web site, or was it a voluntary survey?	See the subsection “Study Design and Participant Recruitment”.<br><br>Voluntary (opt-in). The survey was filled in by individuals that are part of Qualtrics’ online panels.
Incentives	Were any incentives offered (eg, monetary, prizes, or non-monetary incentives such as an offer to provide the survey results)?	See the subsection “Study Design and Participant Recruitment”.<br><br>Participants were compensated approximately $3.75 ($15/hour rate) for participation.
Time/Date	In what timeframe were the data collected?	See the subsection “Study Design and Participant Recruitment”.<br><br>June 17, 2024 - July 26, 2024
Randomization of items or questionnaires	To prevent biases items can be randomized or alternated.	See the subsection “Procedure” and Table 1.<br><br>Each participant viewed six clinical scenarios (lung cancer, sepsis, depression, colon cancer, RSV

		vaccination, and heart failure). Within each scenario, participants were randomly assigned to one of the three decision-source conditions (doctor alone, doctor + risk calculator, doctor + AI), encountering each decision source in two of the six scenarios, with no decision source repeated immediately on consecutive scenarios.
Adaptive questioning	Use adaptive questioning (certain items, or only conditionally displayed based on responses to other items) to reduce number and complexity of the questions.	See Multimedia Appendix 2 (the full survey).<br><br>Used in demographics questions e.g. when participants stated that they are of mixed descent, if they worked as a healthcare professional, and when providing feedback on questions checking participant's comprehension of key concepts.
Number of Items	What was the number of questionnaire items per page? The number of items is an important factor for the completion rate.	See Multimedia Appendix 2 (the full survey).<br><br>The number of items varied, with e.g. the initial demographics questions being provided with up to 6 items per page, while with the main survey items (scenarios with the varying sources) the number of questions was limited to 1-4 per page.
Number of screens (pages)	Over how many pages was the questionnaire distributed? The number of items is an important factor for the completion rate.	See Multimedia Appendix 2 (the full survey).<br><br>The number of screens varied depending on some answers (e.g. if participants stated that they are of mixed descent, an additional page with a question would appear). The minimum number of pages (times when participants must click "next" was 30.).
Completeness check	It is technically possible to do consistency or completeness checks before the questionnaire is submitted. Was this done, and if "yes", how (usually JAVAScript)? An alternative is to check for completeness after the questionnaire has been submitted (and highlight mandatory items).	Respondents could not move forward to the next page without completing mandatory questions. Participants had the opportunity to state Unsure / Prefer Not to Answer for demographic questions, and Neutral to

	If this has been done, it should be reported. All items should provide a non-response option such as “not applicable” or “rather not say”, and selection of one response option should be enforced.	Likert-scale style questions. Participants were informed that they can quit the survey at any time.<br><br>The survey was marked complete only once a respondent has answered all mandatory questions and clicked next on the final page. Then respondents were redirected to a final completion notification page.
Review step	State whether respondents were able to review and change their answers (eg, through a Back button or a Review step which displays a summary of the responses and asks the respondents if they are correct).	Respondents could not return to previous sections of the survey after pressing “next” and moving to a new page.
Unique site visitor	If you provide view rates or participation rates, you need to define how you determined a unique visitor. There are different techniques available, based on IP addresses or cookies or both.	The uniqueness of visitors was checked by Qualtrics’ survey sampling services, and we also recorded an IP address for each participant.
View rate (Ratio of unique survey visitors/unique site visitors)	Requires counting unique visitors to the first page of the survey, divided by the number of unique site visitors (not page views!). It is not unusual to have view rates of less than 0.1 % if the survey is voluntary.	Not applicable.
Participation rate (Ratio of unique visitors who agreed to participate/unique first survey page visitors)	Count the unique number of people who filled in the first survey page (or agreed to participate, for example by checking a checkbox), divided by visitors who visit the first page of the survey (or the informed consents page, if present). This can also be called “recruitment” rate.	Not applicable. Due to the setup of Qualtrics and its survey sampling service, we only received responses from those who consented and completed the survey. Therefore, we do not have access to this data.
Completion rate (Ratio of users who finished the survey/users who agreed to participate)	The number of people submitting the last questionnaire page, divided by the number of people who agreed to participate (or submitted the first survey page). This is only relevant if there is a separate “informed consent” page or if the survey goes over several pages. This is a measure for attrition. Note that “completion” can involve leaving questionnaire items blank. This is not a measure for how completely questionnaires were filled in. (If you need a measure for this, use the word “completeness rate”.)	Not applicable. Due to the setup of Qualtrics and its survey sampling service, we only received responses from those who consented and completed the survey. Therefore, we do not have access to this data. Qualtrics rejected 52 participants due to them failing an attention check, and these participants were replaced with new participants.

Cookies used	Indicate whether cookies were used to assign a unique user identifier to each client computer. If so, mention the page on which the cookie was set and read, and how long the cookie was valid. Were duplicate entries avoided by preventing users access to the survey twice; or were duplicate database entries having the same user ID eliminated before analysis? In the latter case, which entries were kept for analysis (eg, the first entry or the most recent)?	Qualtrics' survey sampling services managed the distribution of the survey and verification of each respondent.<br><br>The sampling service provides users with a unique ID that is used to determine that participants are unique.
IP check	Indicate whether the IP address of the client computer was used to identify potential duplicate entries from the same user. If so, mention the period of time for which no two entries from the same IP address were allowed (eg, 24 hours). Were duplicate entries avoided by preventing users with the same IP address access to the survey twice; or were duplicate database entries having the same IP address within a given period of time eliminated before analysis? If the latter, which entries were kept for analysis (eg, the first entry or the most recent)?	Qualtrics' survey sampling services managed the distribution of the survey and verification of each respondent.<br><br>The sampling service provides users with a unique ID that is used to determine that participants are unique.<br><br>We only received responses from respondents who completed the survey. However, we have access to IP addresses and could verify that there are no duplicates.
Log file analysis	Indicate whether other techniques to analyze the log file for identification of multiple entries were used. If so, please describe.	Using Qualtrics' tools, we checked that no duplicate respondents were identified.
Registration	In "closed" (non-open) surveys, users need to login first and it is easier to prevent duplicate entries from the same user. Describe how this was done. For example, was the survey never displayed a second time once the user had filled it in, or was the username stored together with the survey results and later eliminated? If the latter, which entries were kept for analysis (eg, the first entry or the most recent)?	Qualtrics survey sampling services managed the distribution of the survey and ensured that participants cannot respond twice.
Handling of incomplete questionnaires	Were only completed questionnaires analyzed? Were questionnaires which terminated early (where, for example, users did not go through all questionnaire pages) also analyzed?	See subsection "Participants".<br><br>Only completed responses were saved and analyzed.
Questionnaires submitted with an atypical timestamp	Some investigators may measure the time people needed to fill in a questionnaire and exclude questionnaires that were submitted too soon. Specify the timeframe that was used as a cut-off point, and describe how this point was determined.	Qualtrics' internal tool was used to check that participants are not "speeding" through the survey.

		Responses are flagged for “speeding” when they are more than two standard deviations from the median duration it takes for respondents to take the survey. Screened-out, preview, and test responses are not considered.
Statistical correction	Indicate whether any methods such as weighting of items or propensity scores have been used to adjust for the non-representative sample; if so, please describe the methods.	See subsection “Quantitative Analysis”.<br><br>No. These methods were not utilized in the analysis as we instead utilized quota sampling to ensure balance in the response sample itself.

This checklist has been modified from Eysenbach G. Improving the quality of Web surveys: the Checklist for Reporting Results of Internet E-Surveys (CHERRIES). J Med Internet Res. 2004 Sep 29;6(3):e34 [erratum in J Med Internet Res. 2012; 14(1): e8.]. Article available at https://www.jmir.org/2004/3/e34/; erratum available https://www.jmir.org/2012/1/e8/. Copyright ©Gunther Eysenbach. Originally published in the Journal of Medical Internet Research, 29.9.2004 and 04.01.2012.

This is an open-access article distributed under the terms of the Creative Commons Attribution License (https://creativecommons.org/licenses/by/2.0/), which permits unrestricted use, distribution, and reproduction in any medium, provided the original work, first published in the Journal of Medical Internet Research, is properly cited.
