# Supplementary material for: Investigating Patient Trust and Intention to Act in AI-Supported Clinical Decisions: Cross-Sectional Survey
Source: J Med Internet Res. 2026 Nov 2;28:e100388. doi: 10.2196/100388 (PMC13741335; doi:10.2196/100388)
Supplement: Supplementary file 3 [file jmir-v28-e100388-s002.docx]

**Supplement: Full Regression Data For Preference For Decision Support By Clinical Condition**

Table 1: Whether or not to receive a biopsy for lung cancer

|  | | B | Std. Error | Wald | df | Sig. | Exp(B) | 95% Confidence Interval for Exp(B) | |
| --- | --- | --- | --- | --- | --- | --- | --- | --- | --- |
|  |  |  |  |  |  |  |  | Lower Bound | Upper Bound |
| Doctor/health professional decides alone | Intercept | -0.88 | 0.204 | 18.696 | 1 | 0.000 |  |  |  |
|  | Woman | 0.11 | 0.149 | 0.523 | 1 | 0.470 | 1.114 | 0.832 | 1.491 |
|  | Asian | -0.48 | 0.350 | 1.865 | 1 | 0.172 | 0.620 | 0.312 | 1.232 |
|  | Black | -0.01 | 0.152 | 0.007 | 1 | 0.932 | 0.987 | 0.733 | 1.329 |
|  | Gen X | 0.27 | 0.184 | 2.194 | 1 | 0.139 | 1.312 | 0.916 | 1.881 |
|  | Gen Z | 0.24 | 0.228 | 1.146 | 1 | 0.284 | 1.277 | 0.816 | 1.998 |
|  | Millennials | 0.03 | 0.197 | 0.024 | 1 | 0.877 | 1.031 | 0.701 | 1.517 |
|  | Boomers | 0b |  |  | 0 |  |  |  |  |
|  | Medical Discrimination Low | -0.07 | 0.151 | 0.196 | 1 | 0.658 | 0.935 | 0.695 | 1.258 |
|  | Numeracy Low | -0.07 | 0.149 | 0.230 | 1 | 0.632 | 0.931 | 0.695 | 1.247 |
|  | Literacy Low | 0.22 | 0.152 | 2.115 | 1 | 0.146 | 1.247 | 0.926 | 1.680 |
| Doctor/health professional decides with the help of AI | Intercept | -0.84 | 0.220 | 14.798 | 1 | 0.000 |  |  |  |
|  | Woman | -0.24 | 0.166 | 2.020 | 1 | 0.155 | 0.789 | 0.570 | 1.094 |
|  | Asian | -0.72 | 0.430 | 2.837 | 1 | 0.092 | 0.485 | 0.209 | 1.126 |
|  | Black | -0.08 | 0.170 | 0.243 | 1 | 0.622 | 0.919 | 0.658 | 1.284 |
|  | Gen X | 0.21 | 0.202 | 1.028 | 1 | 0.311 | 1.228 | 0.826 | 1.826 |
|  | Gen Z | -0.03 | 0.265 | 0.013 | 1 | 0.908 | 0.970 | 0.577 | 1.631 |
|  | Millennials | -0.01 | 0.216 | 0.003 | 1 | 0.953 | 0.987 | 0.646 | 1.509 |
|  | Boomers | 0b |  |  | 0 |  |  |  |  |
|  | Medical Discrimination Low | 0.04 | 0.169 | 0.064 | 1 | 0.800 | 1.044 | 0.750 | 1.454 |
|  | Numeracy Low | -0.24 | 0.164 | 2.190 | 1 | 0.139 | 0.784 | 0.568 | 1.082 |
|  | Literacy Low | 0.16 | 0.170 | 0.927 | 1 | 0.336 | 1.177 | 0.844 | 1.642 |

Table 2: Whether or not to recommend a colonoscopy for possible colon cancer

|  | | B | Std. Error | Wald | df | Sig. | Exp(B) | 95% Confidence Interval for Exp(B) | |
| --- | --- | --- | --- | --- | --- | --- | --- | --- | --- |
|  |  |  |  |  |  |  |  | Lower Bound | Upper Bound |
| Doctor/health professional decides alone | Intercept | -0.84 | 0.200 | 17.513 | 1 | 0.000 |  |  |  |
|  | Woman | -0.04 | 0.147 | 0.077 | 1 | 0.781 | 0.960 | 0.719 | 1.281 |
|  | Asian | -0.54 | 0.350 | 2.395 | 1 | 0.122 | 0.582 | 0.293 | 1.155 |
|  | Black | 0.08 | 0.150 | 0.278 | 1 | 0.598 | 1.082 | 0.806 | 1.454 |
|  | Gen X | 0.36 | 0.181 | 4.005 | 1 | 0.045 | 1.435 | 1.007 | 2.045 |
|  | Gen Z | 0.23 | 0.229 | 0.997 | 1 | 0.318 | 1.257 | 0.802 | 1.971 |
|  | Millennials | 0.19 | 0.193 | 0.921 | 1 | 0.337 | 1.204 | 0.824 | 1.757 |
|  | Boomers | 0b |  |  | 0 |  |  |  |  |
|  | Medical Discrimination Low | 0.02 | 0.150 | 0.022 | 1 | 0.881 | 1.023 | 0.763 | 1.371 |
|  | Numeracy Low | 0.16 | 0.147 | 1.153 | 1 | 0.283 | 1.172 | 0.877 | 1.564 |
|  | Literacy Low | 0.01 | 0.150 | 0.003 | 1 | 0.957 | 1.008 | 0.751 | 1.353 |
| Doctor/health professional decides with the help of AI | Intercept | -1.01 | 0.222 | 20.795 | 1 | 0.000 |  |  |  |
|  | Woman | -0.26 | 0.164 | 2.577 | 1 | 0.108 | 0.769 | 0.558 | 1.060 |
|  | Asian | -0.81 | 0.430 | 3.524 | 1 | 0.060 | 0.446 | 0.192 | 1.036 |
|  | Black | -0.02 | 0.167 | 0.018 | 1 | 0.893 | 0.978 | 0.705 | 1.356 |
|  | Gen X | 0.45 | 0.203 | 4.815 | 1 | 0.028 | 1.561 | 1.049 | 2.324 |
|  | Gen Z | 0.32 | 0.255 | 1.617 | 1 | 0.203 | 1.383 | 0.839 | 2.279 |
|  | Millennials | 0.27 | 0.215 | 1.571 | 1 | 0.210 | 1.310 | 0.859 | 1.997 |
|  | Boomers | 0b |  |  | 0 |  |  |  |  |
|  | Medical Discrimination Low | -0.07 | 0.166 | 0.169 | 1 | 0.681 | 0.934 | 0.674 | 1.294 |
|  | Numeracy Low | 0.10 | 0.163 | 0.346 | 1 | 0.556 | 1.100 | 0.800 | 1.514 |
|  | Literacy Low | 0.11 | 0.167 | 0.440 | 1 | 0.507 | 1.117 | 0.805 | 1.549 |

Table 3: Whether or not to send home someone with a low risk of heart failure complications

|  | | B | Std. Error | Wald | df | Sig. | Exp(B) | 95% Confidence Interval for Exp(B) | |
| --- | --- | --- | --- | --- | --- | --- | --- | --- | --- |
|  |  |  |  |  |  |  |  | Lower Bound | Upper Bound |
| Doctor/health professional decides alone | Intercept | -0.81 | 0.200 | 16.619 | 1 | 0.000 |  |  |  |
|  | Woman | -0.04 | 0.145 | 0.086 | 1 | 0.770 | 0.958 | 0.721 | 1.274 |
|  | Asian | -0.85 | 0.371 | 5.184 | 1 | 0.023 | 0.429 | 0.207 | 0.889 |
|  | Black | 0.14 | 0.147 | 0.887 | 1 | 0.346 | 1.149 | 0.861 | 1.534 |
|  | Gen X | 0.42 | 0.178 | 5.599 | 1 | 0.018 | 1.524 | 1.075 | 2.160 |
|  | Gen Z | 0.30 | 0.229 | 1.755 | 1 | 0.185 | 1.355 | 0.865 | 2.122 |
|  | Millennials | 0.26 | 0.191 | 1.790 | 1 | 0.181 | 1.291 | 0.888 | 1.876 |
|  | Boomers | 0b |  |  | 0 |  |  |  |  |
|  | Medical Discrimination Low | 0.00 | 0.147 | 0.000 | 1 | 0.988 | 0.998 | 0.747 | 1.332 |
|  | Numeracy Low | 0.14 | 0.146 | 0.915 | 1 | 0.339 | 1.149 | 0.864 | 1.529 |
|  | Literacy Low | 0.06 | 0.148 | 0.181 | 1 | 0.671 | 1.065 | 0.797 | 1.423 |
| Doctor/health professional decides with the help of AI | Intercept | -0.77 | 0.222 | 12.007 | 1 | 0.001 |  |  |  |
|  | Woman | -0.51 | 0.168 | 9.171 | 1 | 0.002 | 0.600 | 0.432 | 0.835 |
|  | Asian | -0.87 | 0.430 | 4.130 | 1 | 0.042 | 0.418 | 0.180 | 0.969 |
|  | Black | -0.08 | 0.172 | 0.230 | 1 | 0.632 | 0.921 | 0.657 | 1.291 |
|  | Gen X | 0.23 | 0.209 | 1.201 | 1 | 0.273 | 1.257 | 0.835 | 1.893 |
|  | Gen Z | 0.42 | 0.254 | 2.716 | 1 | 0.099 | 1.520 | 0.924 | 2.501 |
|  | Millennials | 0.19 | 0.218 | 0.772 | 1 | 0.379 | 1.211 | 0.790 | 1.858 |
|  | Boomers | 0b |  |  | 0 |  |  |  |  |
|  | Medical Discrimination Low | -0.05 | 0.170 | 0.081 | 1 | 0.776 | 0.953 | 0.682 | 1.330 |
|  | Numeracy Low | 0.09 | 0.166 | 0.268 | 1 | 0.605 | 1.090 | 0.787 | 1.508 |
|  | Literacy Low | -0.02 | 0.171 | 0.021 | 1 | 0.884 | 0.975 | 0.698 | 1.364 |

Table 4: Whether or not to see a psychiatrist for possible depression

|  | | B | Std. Error | Wald | df | Sig. | Exp(B) | 95% Confidence Interval for Exp(B) | |
| --- | --- | --- | --- | --- | --- | --- | --- | --- | --- |
|  |  |  |  |  |  |  |  | Lower Bound | Upper Bound |
| Doctor/health professional decides alone | Intercept | 0.13 | 0.186 | 0.472 | 1 | 0.492 |  |  |  |
|  | Woman | 0.03 | 0.139 | 0.033 | 1 | 0.856 | 1.026 | 0.781 | 1.347 |
|  | Asian | -0.85 | 0.319 | 7.041 | 1 | 0.008 | 0.429 | 0.230 | 0.802 |
|  | Black | 0.15 | 0.143 | 1.153 | 1 | 0.283 | 1.166 | 0.881 | 1.542 |
|  | Gen X | 0.03 | 0.170 | 0.027 | 1 | 0.870 | 1.028 | 0.736 | 1.436 |
|  | Gen Z | -0.06 | 0.216 | 0.069 | 1 | 0.793 | 0.945 | 0.618 | 1.444 |
|  | Millennials | -0.18 | 0.181 | 0.935 | 1 | 0.334 | 0.839 | 0.589 | 1.197 |
|  | Boomers | 0b |  |  | 0 |  |  |  |  |
|  | Medical Discrimination Low | -0.03 | 0.141 | 0.033 | 1 | 0.857 | 0.975 | 0.739 | 1.286 |
|  | Numeracy Low | -0.22 | 0.139 | 2.437 | 1 | 0.118 | 0.805 | 0.612 | 1.057 |
|  | Literacy Low | 0.14 | 0.142 | 0.994 | 1 | 0.319 | 1.152 | 0.872 | 1.522 |
| Doctor/health professional decides with the help of AI | Intercept | -0.86 | 0.247 | 12.194 | 1 | 0.000 |  |  |  |
|  | Woman | -0.24 | 0.180 | 1.789 | 1 | 0.181 | 0.786 | 0.553 | 1.118 |
|  | Asian | -1.19 | 0.497 | 5.773 | 1 | 0.016 | 0.303 | 0.114 | 0.803 |
|  | Black | -0.06 | 0.184 | 0.117 | 1 | 0.732 | 0.939 | 0.655 | 1.347 |
|  | Gen X | 0.48 | 0.226 | 4.424 | 1 | 0.035 | 1.610 | 1.033 | 2.509 |
|  | Gen Z | 0.40 | 0.282 | 2.025 | 1 | 0.155 | 1.494 | 0.860 | 2.596 |
|  | Millennials | 0.31 | 0.238 | 1.743 | 1 | 0.187 | 1.368 | 0.859 | 2.180 |
|  | Boomers | 0b |  |  | 0 |  |  |  |  |
|  | Medical Discrimination Low | 0.02 | 0.183 | 0.010 | 1 | 0.920 | 1.018 | 0.712 | 1.458 |
|  | Numeracy Low | -0.20 | 0.178 | 1.309 | 1 | 0.253 | 0.815 | 0.575 | 1.157 |
|  | Literacy Low | 0.16 | 0.184 | 0.740 | 1 | 0.390 | 1.171 | 0.817 | 1.678 |

Table 5: Whether or not to begin sepsis treatment for your father with heart failure

|  | | B | Std. Error | Wald | df | Sig. | Exp(B) | 95% Confidence Interval for Exp(B) | |
| --- | --- | --- | --- | --- | --- | --- | --- | --- | --- |
|  |  |  |  |  |  |  |  | Lower Bound | Upper Bound |
| Doctor/health professional decides alone | Intercept | -0.75 | 0.199 | 14.251 | 1 | 0.000 |  |  |  |
|  | Woman | 0.19 | 0.146 | 1.657 | 1 | 0.198 | 1.207 | 0.906 | 1.607 |
|  | Asian | -0.77 | 0.358 | 4.646 | 1 | 0.031 | 0.462 | 0.229 | 0.932 |
|  | Black | 0.03 | 0.149 | 0.035 | 1 | 0.851 | 1.028 | 0.769 | 1.376 |
|  | Gen X | 0.34 | 0.179 | 3.670 | 1 | 0.055 | 1.408 | 0.992 | 1.998 |
|  | Gen Z | 0.25 | 0.229 | 1.160 | 1 | 0.282 | 1.279 | 0.817 | 2.003 |
|  | Millennials | -0.02 | 0.192 | 0.011 | 1 | 0.917 | 0.980 | 0.672 | 1.429 |
|  | Boomers | 0b |  |  | 0 |  |  |  |  |
|  | Medical Discrimination Low | -0.06 | 0.148 | 0.144 | 1 | 0.704 | 0.945 | 0.707 | 1.264 |
|  | Numeracy Low | -0.11 | 0.147 | 0.538 | 1 | 0.463 | 0.898 | 0.674 | 1.197 |
|  | Literacy Low | 0.13 | 0.149 | 0.754 | 1 | 0.385 | 1.138 | 0.850 | 1.524 |
| Doctor/health professional decides with the help of AI | Intercept | -0.86 | 0.225 | 14.634 | 1 | 0.000 |  |  |  |
|  | Woman | -0.12 | 0.169 | 0.498 | 1 | 0.480 | 0.887 | 0.637 | 1.236 |
|  | Asian | -1.30 | 0.491 | 6.987 | 1 | 0.008 | 0.273 | 0.104 | 0.715 |
|  | Black | -0.27 | 0.174 | 2.491 | 1 | 0.114 | 0.760 | 0.540 | 1.069 |
|  | Gen X | 0.38 | 0.210 | 3.251 | 1 | 0.071 | 1.460 | 0.968 | 2.202 |
|  | Gen Z | 0.49 | 0.259 | 3.525 | 1 | 0.060 | 1.626 | 0.979 | 2.701 |
|  | Millennials | 0.05 | 0.223 | 0.051 | 1 | 0.822 | 1.052 | 0.679 | 1.629 |
|  | Boomers | 0b |  |  | 0 |  |  |  |  |
|  | Medical Discrimination Low | -0.02 | 0.172 | 0.021 | 1 | 0.884 | 0.975 | 0.696 | 1.366 |
|  | Numeracy Low | -0.34 | 0.167 | 4.257 | 1 | 0.039 | 0.709 | 0.511 | 0.983 |
|  | Literacy Low | 0.21 | 0.173 | 1.517 | 1 | 0.218 | 1.237 | 0.882 | 1.737 |

Table 6: Whether or not to receive a vaccine to protect against RSV (respiratory synthetic virus)

|  | | B | Std. Error | Wald | df | Sig. | Exp(B) | 95% Confidence Interval for Exp(B) | |
| --- | --- | --- | --- | --- | --- | --- | --- | --- | --- |
|  |  |  |  |  |  |  |  | Lower Bound | Upper Bound |
| Doctor/health professional decides alone | Intercept | -0.63 | 0.190 | 10.879 | 1 | 0.001 |  |  |  |
|  | Woman | -0.11 | 0.142 | 0.597 | 1 | 0.440 | 0.896 | 0.679 | 1.183 |
|  | Asian | -0.50 | 0.319 | 2.474 | 1 | 0.116 | 0.606 | 0.324 | 1.131 |
|  | Black | 0.33 | 0.145 | 5.281 | 1 | 0.022 | 1.396 | 1.050 | 1.856 |
|  | Gen X | 0.15 | 0.173 | 0.804 | 1 | 0.370 | 1.167 | 0.832 | 1.637 |
|  | Gen Z | -0.03 | 0.221 | 0.021 | 1 | 0.884 | 0.968 | 0.627 | 1.494 |
|  | Millennials | -0.25 | 0.185 | 1.791 | 1 | 0.181 | 0.780 | 0.543 | 1.122 |
|  | Boomers | 0b |  |  | 0 |  |  |  |  |
|  | Medical Discrimination Low | 0.27 | 0.144 | 3.569 | 1 | 0.059 | 1.314 | 0.990 | 1.743 |
|  | Numeracy Low | 0.16 | 0.142 | 1.265 | 1 | 0.261 | 1.174 | 0.888 | 1.551 |
|  | Literacy Low | 0.16 | 0.145 | 1.244 | 1 | 0.265 | 1.175 | 0.885 | 1.561 |
| Doctor/health professional decides with the help of AI | Intercept | -1.29 | 0.241 | 28.536 | 1 | 0.000 |  |  |  |
|  | Woman | -0.49 | 0.175 | 7.867 | 1 | 0.005 | 0.612 | 0.434 | 0.862 |
|  | Asian | -1.31 | 0.546 | 5.718 | 1 | 0.017 | 0.271 | 0.093 | 0.790 |
|  | Black | 0.22 | 0.177 | 1.591 | 1 | 0.207 | 1.250 | 0.884 | 1.769 |
|  | Gen X | 0.71 | 0.221 | 10.386 | 1 | 0.001 | 2.040 | 1.322 | 3.149 |
|  | Gen Z | 0.64 | 0.270 | 5.631 | 1 | 0.018 | 1.899 | 1.118 | 3.225 |
|  | Millennials | 0.35 | 0.233 | 2.251 | 1 | 0.134 | 1.419 | 0.898 | 2.241 |
|  | Boomers | 0b |  |  | 0 |  |  |  |  |
|  | Medical Discrimination Low | 0.35 | 0.177 | 3.891 | 1 | 0.049 | 1.419 | 1.002 | 2.010 |
|  | Numeracy Low | -0.17 | 0.171 | 0.932 | 1 | 0.334 | 0.847 | 0.606 | 1.186 |
|  | Literacy Low | 0.19 | 0.178 | 1.149 | 1 | 0.284 | 1.210 | 0.854 | 1.714 |
