# Supplementary material for: Investigating Patient Trust and Intention to Act in AI-Supported Clinical Decisions: Cross-Sectional Survey
Source: J Med Internet Res. 2026 Nov 2;28:e100388. doi: 10.2196/100388 (PMC13741335; doi:10.2196/100388)
Supplement: Supplementary file 4 [file jmir-v28-e100388-s003.docx]

**Supplement: Additional Numerical Data from Figures and Subgroup Sample Sizes**

**1	Figure 1, Figure 2, and Figure 3 numerical data**

Table 1:	Numerical data for Figure 1. Behavioral intentions across clinical conditions

| Condition | Source | Likely | Undecided | Unlikely |
| --- | --- | --- | --- | --- |
| Colon Cancer | Doctor + AI | 270 | 61 | 49 |
| Colon Cancer | Doctor + Risk Calculator | 277 | 60 | 61 |
| Colon Cancer | Doctor | 281 | 61 | 64 |
| Depression | Doctor + AI | 216 | 79 | 111 |
| Depression | Doctor + Risk Calculator | 207 | 80 | 94 |
| Depression | Doctor | 205 | 72 | 121 |
| Heart Failure | Doctor + AI | 184 | 92 | 122 |
| Heart Failure | Doctor + Risk Calculator | 216 | 70 | 119 |
| Heart Failure | Doctor | 178 | 85 | 118 |
| Lung Cancer | Doctor + AI | 269 | 70 | 59 |
| Lung Cancer | Doctor + Risk Calculator | 299 | 58 | 49 |
| Lung Cancer | Doctor | 286 | 59 | 36 |
| RSV | Doctor + AI | 250 | 69 | 87 |
| RSV | Doctor + Risk Calculator | 249 | 70 | 62 |
| RSV | Doctor | 253 | 84 | 61 |
| Sepsis | Doctor + AI | 264 | 74 | 43 |
| Sepsis | Doctor + Risk Calculator | 286 | 70 | 42 |
| Sepsis | Doctor | 285 | 63 | 58 |

Table 2:	Numerical data for Figure 2. Trust across clinical conditions

| Condition | Source | Agree | Neither agree nor disagree | Disagree |
| --- | --- | --- | --- | --- |
| Colon Cancer | Doctor + AI | 251 | 68 | 62 |
| Colon Cancer | Doctor + Risk Calculator | 274 | 78 | 46 |
| Colon Cancer | Doctor | 322 | 44 | 40 |
| Depression | Doctor + AI | 207 | 78 | 121 |
| Depression | Doctor + Risk Calculator | 222 | 81 | 78 |
| Depression | Doctor | 222 | 92 | 84 |
| Heart Failure | Doctor + AI | 200 | 76 | 122 |
| Heart Failure | Doctor + Risk Calculator | 254 | 61 | 91 |
| Heart Failure | Doctor | 247 | 53 | 81 |
| Lung Cancer | Doctor + AI | 260 | 72 | 66 |
| Lung Cancer | Doctor + Risk Calculator | 301 | 50 | 55 |
| Lung Cancer | Doctor | 316 | 39 | 26 |
| RSV | Doctor + AI | 256 | 67 | 83 |
| RSV | Doctor + Risk Calculator | 256 | 65 | 60 |
| RSV | Doctor | 276 | 69 | 53 |
| Sepsis | Doctor + AI | 236 | 64 | 81 |
| Sepsis | Doctor + Risk Calculator | 268 | 71 | 59 |
| Sepsis | Doctor | 310 | 46 | 50 |

Table 3:	Numerical data for Figure 3. Preference for decision support by clinical condition*

| Condition | Preference | n | Proportion |
| --- | --- | --- | --- |
| Lung Cancer | Doctor/health professional decides alone | 312 | 0.267 |
| Lung Cancer | Doctor/health professional decides with the help of a risk calculator | 627 | 0.536 |
| Lung Cancer | Doctor/health professional decides with the help of AI | 230 | 0.197 |
| Colon Cancer | Doctor/health professional decides alone | 334 | 0.286 |
| Colon Cancer | Doctor/health professional decides with the help of a risk calculator | 588 | 0.503 |
| Colon Cancer | Doctor/health professional decides with the help of AI | 247 | 0.211 |
| Heart Failure | Doctor/health professional decides alone | 358 | 0.306 |
| Heart Failure | Doctor/health professional decides with the help of a risk calculator | 576 | 0.493 |
| Heart Failure | Doctor/health professional decides with the help of AI | 235 | 0.201 |
| Depression | Doctor/health professional decides alone | 492 | 0.421 |
| Depression | Doctor/health professional decides with the help of a risk calculator | 468 | 0.400 |
| Depression | Doctor/health professional decides with the help of AI | 209 | 0.179 |
| Sepsis | Doctor/health professional decides alone | 339 | 0.290 |
| Sepsis | Doctor/health professional decides with the help of a risk calculator | 604 | 0.517 |
| Sepsis | Doctor/health professional decides with the help of AI | 226 | 0.193 |
| RSV | Doctor/health professional decides alone | 408 | 0.349 |
| RSV | Doctor/health professional decides with the help of a risk calculator | 540 | 0.462 |
| RSV | Doctor/health professional decides with the help of AI | 221 | 0.189 |

* Note that 16 participants were excluded from the statistical analysis due to choosing to provide the responses “Prefer Not To Answer” to specific demographic questions.

**2	Subgroup sample sizes by condition.**

Table 4: Colon Cancer subgroup sample sizes

|  | Doctor | Doctor + AI | Doctor + Risk Calculator |
| --- | --- | --- | --- |
| Women | 205 | 191 | 202 |
| Asian | 21 | 13 | 23 |
| Black/African American | 172 | 154 | 164 |
| Gen X | 117 | 112 | 108 |
| Millennial | 99 | 93 | 100 |
| Gen Z | 60 | 53 | 54 |
| Literacy Low | 223 | 199 | 202 |
| Numeracy Low | 229 | 221 | 224 |
| Med. Discrimination Low | 211 | 202 | 191 |
| All | 406 | 380 | 398 |

Table 5: Depression subgroup sample sizes

|  | Doctor | Doctor + AI | Doctor + Risk Calculator |
| --- | --- | --- | --- |
| Women | 202 | 205 | 192 |
| Asian | 23 | 21 | 13 |
| Black/African American | 164 | 172 | 155 |
| Gen X | 108 | 117 | 112 |
| Millennial | 100 | 99 | 93 |
| Gen Z | 54 | 60 | 54 |
| Literacy Low | 202 | 223 | 200 |
| Numeracy Low | 224 | 229 | 222 |
| Med. Discrimination Low | 191 | 211 | 202 |
| All | 398 | 406 | 381 |

Table 6: Heart Failure subgroup sample sizes

|  | Doctor | Doctor + AI | Doctor + Risk Calculator |
| --- | --- | --- | --- |
| Women | 192 | 202 | 205 |
| Asian | 13 | 23 | 21 |
| Black/African American | 155 | 164 | 171 |
| Gen X | 112 | 108 | 116 |
| Millennial | 93 | 100 | 99 |
| Gen Z | 54 | 54 | 60 |
| Literacy Low | 200 | 202 | 223 |
| Numeracy Low | 222 | 224 | 229 |
| Med. Discrimination Low | 202 | 191 | 211 |
| All | 381 | 398 | 405 |

Table 7: Lung Cancer subgroup sample sizes

|  | Doctor | Doctor + AI | Doctor + Risk Calculator |
| --- | --- | --- | --- |
| Women | 192 | 202 | 205 |
| Asian | 13 | 23 | 21 |
| Black/African American | 155 | 164 | 172 |
| Gen X | 112 | 108 | 117 |
| Millennial | 93 | 100 | 99 |
| Gen Z | 54 | 54 | 60 |
| Literacy Low | 200 | 202 | 223 |
| Numeracy Low | 222 | 224 | 229 |
| Med. Discrimination Low | 202 | 191 | 211 |
| All | 381 | 398 | 406 |

Table 8: RSV subgroup sample sizes

|  | Doctor | Doctor + AI | Doctor + Risk Calculator |
| --- | --- | --- | --- |
| Women | 202 | 205 | 192 |
| Asian | 23 | 21 | 13 |
| Black/African American | 164 | 172 | 155 |
| Gen X | 108 | 117 | 112 |
| Millennial | 100 | 99 | 93 |
| Gen Z | 54 | 60 | 54 |
| Literacy Low | 202 | 223 | 200 |
| Numeracy Low | 224 | 229 | 222 |
| Med. Discrimination Low | 191 | 211 | 202 |
| All | 398 | 406 | 381 |

Table 9: Sepsis subgroup sample sizes

|  | Doctor | Doctor + AI | Doctor + Risk Calculator |
| --- | --- | --- | --- |
| Women | 205 | 192 | 202 |
| Asian | 21 | 13 | 23 |
| Black/African American | 172 | 155 | 164 |
| Gen X | 117 | 112 | 108 |
| Millennial | 99 | 93 | 100 |
| Gen Z | 60 | 54 | 54 |
| Literacy Low | 223 | 200 | 202 |
| Numeracy Low | 229 | 222 | 224 |
| Med. Discrimination Low | 211 | 202 | 191 |
| All | 406 | 381 | 398 |

**3	Subgroup sample sizes for Figure 3 (preferences)**

Table 10: Colon Cancer subgroup sample sizes

|  | Doctor | Doctor + AI | Doctor + Risk Calculator |
| --- | --- | --- | --- |
| Women | 181 | 306 | 112 |
| Asian | 13 | 37 | 7 |
| Black/African American | 151 | 237 | 103 |
| Gen X | 108 | 151 | 78 |
| Millennial | 86 | 142 | 64 |
| Gen Z | 49 | 82 | 37 |
| Literacy Low | 180 | 326 | 119 |
| Numeracy Low | 204 | 330 | 141 |
| Med. Discrimination Low | 175 | 311 | 118 |

Table 11: Depression subgroup sample sizes

|  | Doctor | Doctor + AI | Doctor + Risk Calculator |
| --- | --- | --- | --- |
| Women | 262 | 244 | 93 |
| Asian | 17 | 35 | 5 |
| Black/African American | 221 | 185 | 85 |
| Gen X | 145 | 124 | 68 |
| Millennial | 118 | 117 | 57 |
| Gen Z | 70 | 66 | 32 |
| Literacy Low | 267 | 255 | 103 |
| Numeracy Low | 280 | 281 | 114 |
| Med. Discrimination Low | 255 | 246 | 103 |

Table 12: Lung Cancer subgroup sample sizes

|  | Doctor | Doctor + AI | Doctor + Risk Calculator |
| --- | --- | --- | --- |
| Women | 173 | 326 | 100 |
| Asian | 13 | 37 | 7 |
| Black/African American | 139 | 263 | 89 |
| Gen X | 101 | 167 | 69 |
| Millennial | 79 | 157 | 56 |
| Gen Z | 50 | 89 | 29 |
| Literacy Low | 162 | 348 | 115 |
| Numeracy Low | 187 | 370 | 118 |
| Med. Discrimination Low | 154 | 330 | 120 |
|  |  |  |  |

Table 13: Heart Failure subgroup sample sizes

|  | Doctor | Doctor + AI | Doctor + Risk Calculator |
| --- | --- | --- | --- |
| Women | 198 | 306 | 95 |
| Asian | 11 | 39 | 7 |
| Black/African American | 170 | 229 | 92 |
| Gen X | 120 | 151 | 66 |
| Millennial | 95 | 138 | 59 |
| Gen Z | 53 | 76 | 39 |
| Literacy Low | 189 | 312 | 124 |
| Numeracy Low | 221 | 324 | 130 |
| Med. Discrimination Low | 182 | 305 | 117 |

Table 14: Sepsis subgroup sample sizes

|  | Doctor | Doctor + AI | Doctor + Risk Calculator |
| --- | --- | --- | --- |
| Women | 192 | 308 | 99 |
| Asian | 12 | 40 | 5 |
| Black/African American | 157 | 249 | 85 |
| Gen X | 113 | 154 | 70 |
| Millennial | 83 | 155 | 54 |
| Gen Z | 51 | 80 | 37 |
| Literacy Low | 185 | 331 | 109 |
| Numeracy Low | 204 | 357 | 114 |
| Med. Discrimination Low | 172 | 320 | 112 |

Table 15: RSV subgroup sample sizes

|  | Doctor | Doctor + AI | Doctor + Risk Calculator |
| --- | --- | --- | --- |
| Women | 223 | 286 | 90 |
| Asian | 17 | 36 | 4 |
| Black/African American | 193 | 202 | 96 |
| Gen X | 127 | 137 | 73 |
| Millennial | 91 | 144 | 57 |
| Gen Z | 59 | 71 | 38 |
| Literacy Low | 225 | 290 | 110 |
| Numeracy Low | 256 | 306 | 113 |
| Med. Discrimination Low | 224 | 264 | 116 |
