# Supplementary material for: Investigating Patient Trust and Intention to Act in AI-Supported Clinical Decisions: Cross-Sectional Survey
Source: J Med Internet Res. 2026 Nov 2;28:e100388. doi: 10.2196/100388 (PMC13741335; doi:10.2196/100388)
Supplement: Supplementary file 5 [file jmir-v28-e100388-s004.docx]

**Supplement: Claude Transcripts**

Transcript 1:

Transcript 2:

Transcript 3:

Links to these Claude discussions are available upon request.
